# Genome-wide polygenic risk scores for colorectal cancer have implications for risk-based screening

**DOI:** 10.1038/s41416-023-02536-z

**Published:** 2024-01-03

**Authors:** Max Tamlander, Bradley Jermy, Toni T. Seppälä, Martti Färkkilä, FinnGen, Elisabeth Widén, Samuli Ripatti, Nina Mars

**Affiliations:** 1 Institute for Molecular Medicine Finland, FIMM, HiLIFE, University of Helsinki, Helsinki, Finland; 2 Faculty of Medicine and Health Technology, University of Tampere and TAYS Cancer Centre, Tampere, Finland; 3 Department of Gastroenterology and Alimentary Tract Surgery, Tampere University Hospital, Tampere, Finland; 4 Applied Tumor Genomics Research Program, University of Helsinki, Helsinki, Finland; 5 Abdominal Center, Helsinki University Hospital, Helsinki University, Helsinki, Finland; 6 Clinicum, Department of Public Health, University of Helsinki, Helsinki, Finland; 7 Broad Institute of MIT and Harvard, Cambridge, MA USA

**Keywords:** Preventive medicine, Colorectal cancer, Predictive markers

## Abstract

**Background:**

Hereditary factors, including single genetic variants and family history, can be used for targeting colorectal cancer (CRC) screening, but limited data exist on the impact of polygenic risk scores (PRS) on risk-based CRC screening.

**Methods:**

Using longitudinal health and genomics data on 453,733 Finnish individuals including 8801 CRC cases, we estimated the impact of a genome-wide CRC PRS on CRC screening initiation age through population-calibrated incidence estimation over the life course in men and women.

**Results:**

Compared to the cumulative incidence of CRC at age 60 in Finland (the current age for starting screening in Finland), a comparable cumulative incidence was reached 5 and 11 years earlier in persons with high PRS (80–99% and >99%, respectively), while those with a low PRS (< 20%) reached comparable incidence 7 years later. The PRS was associated with increased risk of post-colonoscopy CRC after negative colonoscopy (hazard ratio 1.76 per PRS SD, 95% CI 1.54–2.01). Moreover, the PRS predicted colorectal adenoma incidence and improved incident CRC risk prediction over non-genetic risk factors.

**Conclusions:**

Our findings demonstrate that a CRC PRS can be used for risk stratification of CRC, with further research needed to optimally integrate the PRS into risk-based screening.

## Background

Colorectal cancer (CRC) is the third most diagnosed cancer and the second leading cause of cancer mortality worldwide [1], making it an appealing focus for population-wide screening efforts [2, 3]. Early and timely colonoscopy screening is particularly beneficial for individuals at elevated risk due to family history of the disease [4] or with the presence of high- or moderate-impact pathogenic variants in CRC susceptibility genes [5–7], such as those affecting DNA mismatch repair (*MLH1, MSH2, MSH6, PMS2*) in Lynch syndrome.

In addition to inherited predisposition captured by family history or clinical multigene panel testing for inherited cancer syndromes, recent advances in genome-wide association studies [8–11] have identified hundreds of common-variant associations for CRC, demonstrating a strong and polygenic pattern of inheritance. While initial analyses suggest that combining these common genome-wide genetic effects into a polygenic risk score (PRS) identifies individuals at elevated disease risk [12–17], accurate population-calibrated estimates of lifetime risks are needed for incorporation of PRS into risk-based screening. Furthermore, data on the impact of CRC PRSs on key drivers and characteristics of the disease, such as precursor adenomas [16, 18], sex- and site-specific disparities [19–22], and risk of subsequent CRC after negative findings in colonoscopy (post-colonoscopy CRC) [23, 24], are still scarce. Here, we built a genome-wide PRS for CRC and performed careful calibration leveraging nationwide cancer registry data for population-specific cumulative incidence estimates, to quantify optimal PRS-informed CRC screening ages in the Finnish population. Using the FinnGen study [25] comprising 453,733 Finnish individuals, we (1) evaluated the performance of the PRS in the context of population-level screening for CRC, (2) assessed the impact of the PRS on post-colonoscopy CRC and (3) tested how the PRS impacts clinical characteristics and clinical risk prediction of CRC.

## Methods

### Study population

FinnGen Data Freeze 11 with 453,733 Finnish individuals comprises prospective epidemiological and disease-based cohorts and hospital biobank samples (Supplementary Table 1). The data were linked by national personal identification numbers to national registries, including the Finnish Cancer Registry (available from 1953 onwards, coverage for CRC exceeding 97% [26, 27]), and national hospital discharge (inpatient visits 1969–, outpatient visits 1998–) and death (1964–) registries. FinnGen Data Freeze 11 used in this study comprised 8801 cases of CRC, with 3.4 million person-years of follow-up time available since study recruitment.

### Polygenic risk scores

We built a genome-wide PRS for CRC using the software PRS-CS [28] (PRS-CS-auto, with 1000 Genomes Project [29] European sample, *N*  =  503, as the external linkage disequilibrium reference panel) using HapMap3 variants. The PRS-CS algorithm utilises a Bayesian regression framework for posterior inference of SNP effect sizes, and we chose PRS-CS over alternative genome-wide PRS development approaches as it enables precise multivariate modelling of linkage disequilibrium in polygenic prediction alongside computational advantages. We used the full summary statistics from a large European ancestry CRC genome-wide association study [9] with 78,473 CRC cases and 107,143 controls. The CRC PRS variant count was 1,088,133. A small number of CRC cases (*N* = 147) and CRC-free individuals in FinnGen (*N* = 8296) were included in the discovery genome-wide association study. As we are unable to identify these exact individuals in FinnGen, we performed a sensitivity analysis by exclusion based on genotyping array information, which did not impact our PRS effect size (Supplementary Table 2).

For comparison to our PRS-CS score, we calculated a previously published 205 single-nucleotide polymorphism score [9] (PRS_205_, with 183 variants available and polymorphic in FinnGen), which the PRS-CS score outperformed (Supplementary Table 3). Our PRSs showed acceptable goodness-of-fit, which we assessed using R package survMisc (Supplementary Fig. 1). To test a PRS independent of Lynch syndrome variants, we performed a supplementary analysis excluding 60,656 single-nucleotide polymorphisms within ±2 megabases of *MLH1, MSH2, MSH6* and *PMS2* from the full discovery genome-wide association study summary statistics before applying PRS-CS (Supplementary Table 3).

### Outcomes and risk factor definitions

We ascertained disease cases using national registries. CRC cases were identified through the Finnish Cancer Registry with International Classification of Diseases for Oncology, 3rd Edition (ICD-O-3) codes C18–C20 and from the death registry with ICD-10 codes C18–C20, or ICD-9 codes of 153, 1540 and 154, or ICD-8 codes of 153, 1540 and 1541. Ascertainment for colorectal adenomas, including those presenting with high-grade dysplasia, was based on ICD and ICD-O-3 codes. For site-specific analyses, we defined proximal colon as constituting the caecum, ascending colon, hepatic flexure, and transverse colon, and the distal colon as constituting the splenic flexure, descending colon and rectosigmoid junction. We used ICD-O-3 morphology codes in the Finnish Cancer Registry data to identify CRCs which were histologically either adenocarcinoma or any other histological subtype. We also separately analysed CRC cases by spread at presentation (a Finnish Cancer Registry classification for localised vs non-localised cancer) and early-onset (age < 50) and late-onset (age ≥ 50) CRC cases.

For post-colonoscopy CRC ascertainment, we identified clinically average-risk (details in Supplementary Information) individuals who had undergone colonoscopy for any indication and were at least 40 years of age at the date of the index examination and who did not have previously diagnosed CRC or a diagnosis of colorectal adenoma within three months before or after the date of the index colonoscopy in electronic health records. These individuals were followed in the Finnish Cancer Registry and death registry data for the occurrence of post-colonoscopy CRC diagnosed 6 months to 10 years after the index colonoscopy. Detailed endpoint and risk factor definitions are described in Supplementary Information and Supplementary Data 1.

### Statistical analysis

We used adjusted Cox proportional hazards models to estimate HRs and 95% CIs for the PRSs, with age as the baseline timescale in the models except for the post-colonoscopy CRC and incident disease analyses, as described below. The proportional hazards assumption was met when tested with scaled Schoenfeld residuals and log-log inspection. In CRC and colorectal adenoma lifetime risk analysis, we used Cox proportional hazards models to estimate sex-specific HRs and 95% CIs with age at disease onset as the timescale, estimating the impact of PRS on CRC separately among men and women. The following PRS categories were primarily applied: <20%, 20–80% (reference), 80–99% or >99% (in CRC), <1%, 1–20%, 20–80% (reference), 80–99% or >99% (in adenoma analysis), and the Cox regression models were adjusted for the first 10 genetic principal components of ancestry, genotyping batch and subcohort. These default groupings were selected to demonstrate the impact of high versus low PRS on absolute risk of CRC and reaching of CRC lifetime risk incidence corresponding to screening onset at age 60 in Finland. Follow-up ended at the age of first record of CRC (in CRC analysis) or colorectal adenoma (in adenoma analysis), age at death, or age at the censoring date 2 November 2022 or at age 80, whichever came first. In post-colonoscopy CRC analysis, we used Cox model with standardised PRS (both with categories <10%, 10–90%, >90% and on the continuous scale), adjusted for the first ten genetic principal components of ancestry, genotyping batch, subcohort, and age at the index colonoscopy. We could not utilise the default groupings in post-colonoscopy CRC analysis due to a smaller sample size.

To compute the lifetime risk of CRC (the probability of developing CRC from birth up to the age of 80 while accounting for the competing risk of death from other causes than CRC), we utilised sex-specific estimates of age-specific (for 5-year age groups) incidence, prevalence, and mortality for Finland included in the Global Burden of Disease (GBD) 2019 data [30], following a risk calibration approach detailed in Jermy et al. [31]. In line with a recently published study on lifetime risk of CRC in patients with Lynch syndrome [32], we assessed lifetime risk of CRC by age 80.

Survival curves were estimated using a lifetime risk approach as detailed above or Kaplan–Meier survival curves using R package survminer. All statistical tests were two-sided and a P value of less than 0.05 was considered to indicate statistical significance. All statistical analyses were performed using R (version 4.2.3).

## Results

### Demographic and PRS characteristics

First, we developed a genome-wide PRS for CRC using the PRS-CS algorithm [28] and weights from a large European genome-wide association study [9]. The PRS was constructed by summing the weights of single-nucleotide polymorphisms while accounting for linkage disequilibrium. PRS performance was evaluated within the FinnGen study (*N* = 453,733; 56.1% women), with the full data containing 8801 CRC cases and 28,200 colorectal adenoma cases. Baseline characteristics of the study participants are shown in Supplementary Table 4.

Individuals with a high PRS were at elevated lifetime risk for CRC by age 80, during which 3245 women and 4380 men were diagnosed with CRC. The adjusted hazard ratio (HR) per standard deviation (SD) increment in the PRS was 1.64 (95% confidence interval [CI] 1.60–1.68, *P* < 1.00 × 10^−300^) for CRC. Compared to those with an average PRS (20–80th percentiles) of the PRS distribution, those in the highest 80–99th and >99th percentiles of the distribution had sex-specific adjusted HRs of 1.93 (1.79–2.08, *P* = 1.18 × 10^−62^) and 3.62 (2.98–4.40, *P* = 7.84 × 10^−38^) in women and 2.01 (1.88–2.14, *P* = 1.63 × 10^−96^) and 3.87 (3.28–4.57, *P* = 4.13 × 10^−58^) in men, respectively. Conversely, those in the lowest 20% had HR estimates of 0.63 (0.56–0.70, *P* = 7.56 × 10^−16^) in women and 0.51 (0.45–0.56, *P* = 1.61 × 10^−36^) in men. Effect sizes for proximal and distal colon and rectal cancer are shown in Supplementary Data 2. Overall, the HR estimates showed a pattern of larger effect sizes for men as compared to women and for distal CRC as compared to cancers of the proximal colon.

### PRS and lifetime risks of CRC and adenomas

We calculated sex- and population-specific estimates of cumulative incidence by PRS groups (PRS < 20%, 20–80%, 80–99% and >99%) in the Finnish population. To achieve this, we used the adjusted HR estimates for CRC and calibrated the baseline risk using Finnish population-based data drawn from the nationwide Finnish Cancer Registry, accounting for age- and sex-specific effects.

**Fig. 1:**
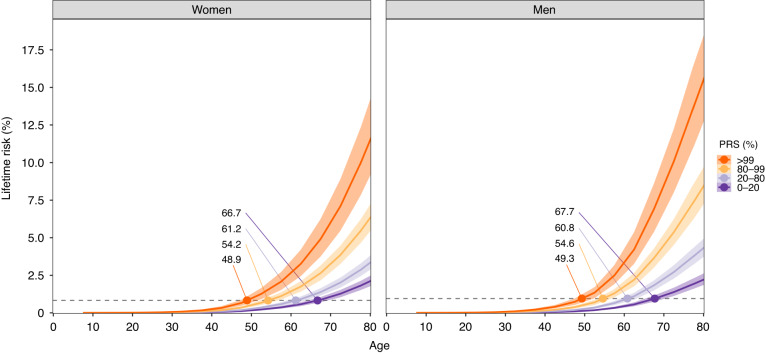
Lifetime risk of CRC and recommended CRC screening initiation age according to polygenic risk score (PRS) in men and women. The dashed line indicates the cumulative incidence for the population average for 60-year-old women (0.83%) and men (0.95%) in the Finnish population. The coloured points with respective numbers show the age at which each polygenic risk score (PRS) category reaches the same cumulative incidence as the average population. The shaded bands represent 95% confidence intervals.

Figure 1 shows the sex-specific lifetime risks of CRC according to the PRS groups. At age 60, when biennial CRC screening with faecal immunochemical testing currently starts in Finland [33, 34], the population cumulative incidences were estimated at 0.83% in women and 0.95% in men (Supplementary Fig. 2). The individuals with an average PRS (20–80th percentile) reached this level at age 60.8 (men) and 61.2 (women). As compared to individuals with an average PRS, individuals with a high PRS in the 80–99th percentile reached the same cumulative risks 6.2 (men) and 7.0 (women) years earlier, and up to 11.5 (men) and 12.3 (women) years earlier among those with a PRS in the 99th percentile. Conversely, those with a low PRS (below the 20th percentile) reached the same cumulative incidence 6.9 (men) and 5.5 (women) years later. Similar patterns were observed at other common CRC screening initiation thresholds, such as 45, 50 and 55 years of age, which are recommended for average-risk individuals in screening guidelines both within the United States [35] and the European Union [36] (Table 1). The lifetime risks of CRC by age 80 were higher among men compared to women, with the largest differences emerging after age 60. With average PRS (20–80th percentile), the lifetime risks were estimated at 4.3% (95% CI 3.7–4.9%) in men and 3.3% (2.9–3.8%) in women. In comparison, the lifetime risk for men with a PRS of 80–99th and >99th percentiles were estimated at 8.4% (7.2–9.7%) and 15.5% (12.6–18.5%), respectively, and for men below the 20th percentile of the PRS, at 2.2% (95% CI 1.9–2.6%). Among women, the corresponding risks were 6.3% (95% CI 5.4–7.2%), 11.5% (9.2–14.3%), respectively, in the 80–99th and >99th percentiles, and 2.1% (1.8–2.5%) below the 20th percentile of the PRS. The cumulative incidences by PRS deciles are in Supplementary Fig. 3.

**Table 1:** Recommended CRC screening initiation age by polygenic risk score (PRS) category at different age thresholds for men and women.

Screening initiation age	PRS-informed screening initiation age (95% CI)	
	Women	Men
At age 45 y		
PRS > 99%	33.6 (31.5–36.0)	32.9 (31.0–35.0)
PRS 80–99%	38.1 (36.3–40.2)	37.6 (35.5–39.6)
PRS 20–80%	43.0 (41.3–44.8)	43.0 (41.1–45.2)
PRS 0–20%	46.3 (44.5–48.3)	48.5 (46.7–51.1)
At age 50 y		
PRS > 99%	41.5 (39.1–43.8)	39.7 (37.6–42.6)
PRS 80–99%	45.9 (44.3–48.1)	45.1 (43.4–47.8)
PRS 20–80%	50.9 (49.4–53.1)	50.8 (49.0–53.1)
PRS 0–20%	55.2 (53.2–57.9)	56.4 (54.4–58.6)
At age 55 y		
PRS > 99%	45.4 (43.4–47.9)	44.7 (42.8–47.7)
PRS 80–99%	50.1 (48.6–52.3)	50.1 (48.4–52.6)
PRS 20–80%	56.2 (54.2–58.5)	55.8 (54.1–58.0)
PRS 0–20%	61.1 (59.0–63.8)	62.2 (59.8–64.2)
At age 60 y		
PRS > 99%	48.9 (47.0–51.2)	49.3 (47.5–52.2)
PRS 80–99%	54.2 (52.6–56.7)	54.6 (53.1–56.9)
PRS 20–80%	61.2 (59.2–63.5)	60.8 (58.9–63.0)
PRS 0–20%	66.7 (64.4–69.3)	67.7 (65.4–70.2)

The age thresholds represent the age when individuals in the PRS bin reach the same cumulative incidence for the population average at the selected age. Results for age 60 correspond to Fig. 1, with results shown for three additional younger screening initiation ages (45, 50 and 55 years). The 95% confidence intervals represent the 2.5th and 97.5th percentiles around the point estimates. The population average estimates were derived using incidences at the screening initiation ages in the Finnish population among men and women (Supplementary Fig. 2).

**Fig. 2:**
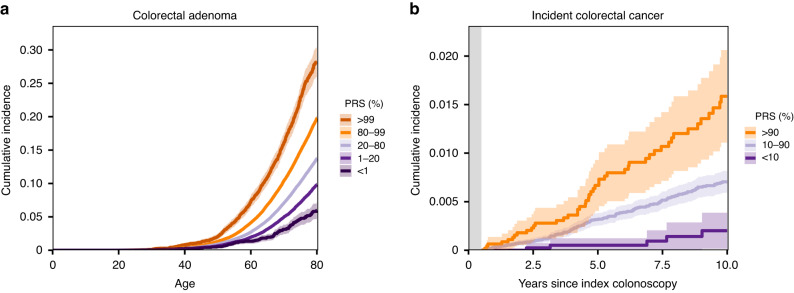
Cumulative incidence for colorectal adenomas and post-colonoscopy CRC according to polygenic risk score (PRS). Cumulative risk of **a** colorectal adenomas (*N* = 453,733; 25,988 cases by age 80) and **b** 10-year incident CRC after negative colonoscopy (*N* = 48,951; 214 cases) by polygenic risk score (PRS) category. **a** Shows the cumulative risk for colorectal adenoma among individuals who had PRS in one of the following categories: less than 1%, 1–20%, 20–80%, 80–99% and above 99%. **b** Shows cumulative incidence for CRC among clinically average-risk individuals who underwent colonoscopy at 40 years of age or older for any indication and had PRS in one of the following categories: less than 10%, 10 to 90%, and above 90%. The shaded bands represent 95% confidence intervals. **b** The grey vertical bar represents the first 6 months after index colonoscopy as we only included cases diagnosed 6 months to 10 years after colonoscopy. CRC colorectal cancer.

In addition to CRC, we observed a similar cumulative incidence pattern for colorectal adenomas with the CRC PRS (Fig. 2a), with 12,920 and 13,068 cases by age 80 in women and men, respectively. The lifetime adenoma risks ranged from 6.0% (95% CI 4.8–7.2%) in the lowest PRS percentile to 28.2% (25.9–30.3%) in the highest percentile, compared to individuals with an average PRS (20–80th percentiles) with the lifetime risk of 13.7% (95% CI 13.5–13.9%). The cumulative incidences among individuals who had undergone colonoscopy are shown in Supplementary Fig. 4. The covariate-adjusted PRS effect sizes for colorectal adenomas are in Supplementary Data 2.

### PRS and risk of post-colonoscopy CRC

For post-colonoscopy CRC analysis, we identified 48,638 clinically average-risk individuals (60.3% women) who underwent colonoscopy for any indication at 40 years of age or older and were followed through cancer and death registry data after a negative colonoscopy for a median of 80.6 months (interquartile range [IQR] 39.6–120.0), during which 214 individuals were diagnosed with post-colonoscopy CRC. The mean age at index colonoscopy was 62.5 (IQR 53.4–71.3). The median interval between the index colonoscopy and diagnosis of post-colonoscopy CRC was 54.7 months (IQR 36.9–83.5 months). The adjusted continuous HR per one SD change in the PRS was 1.76 for post-colonoscopy CRC (95% CI 1.54–2.01, *P* = 2.36 × 10^–16^). Those with a high PRS above the 90th percentile compared to those with an average or low PRS (10–90th percentile and below the 10th percentile of the distribution) were at elevated risk of post-colonoscopy CRC (Fig. 2b). The adjusted HRs for post-colonoscopy CRC for the high and low PRS groups as compared to the average PRS group were 2.23 (95% CI 1.61–3.09, *P* = 1.51 × 10^–6^) and 0.23 (95% CI 0.095–0.56, *P* = 0.0012), respectively. Distributions of PRS and age at index colonoscopy by post-colonoscopy CRC status are shown in Supplementary Fig. 5.

### PRS, clinical characteristics and clinical risk prediction

**Fig. 3:**
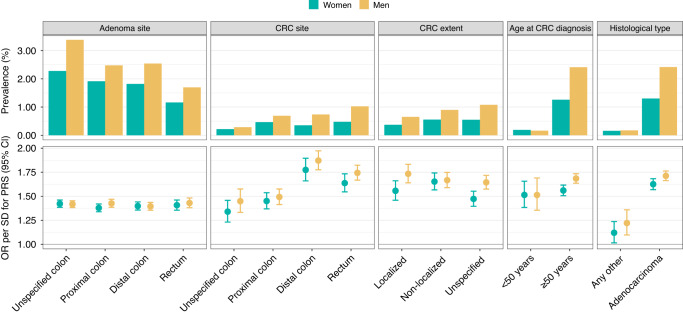
Prevalence and odds ratio (OR) per standard deviation (SD) for the colorectal cancer (CRC) PRS for CRC and colorectal adenomas and among women (*N* = 254,618) and men (*N* = 199,115). The grey boxes represent the disease characteristic category. Total sample size *N* = 453,733. ORs were obtained from logistic regression models adjusted for sex, birth year, genotyping array, subcohort and the first ten genetic principal components of ancestry. Localised extent definition includes localised and locally advanced tumours. Non-localised extent definition includes tumours metastasised to local or distal lymph nodes and distant metastasis.

Lastly, we assessed the impact of the PRS on clinical characteristics of CRC, and its relative performance in clinical risk prediction of CRC. The PRS had a higher adjusted odds ratio (OR) per SD for cancers of the distal colorectum compared to cancers of the proximal colon in both sexes (Fig. 3). In contrast to invasive cancer, preinvasive colorectal adenomas did not show a similar disparity in distal versus proximal anatomical site. Overall, among men and women combined, the OR per SD in the PRS for CRC was 1.62 (95% CI, 1.59–1.65), and 1.57 (95% CI, 1.53–1.62) for colon cancer and 1.70 (95% CI, 1.64–1.76) for rectal cancer. The effect size in women was lower than in men for overall CRC (interaction term *P* = 0.0014) and colon cancer (*P* = 0.013), but with no difference in rectal cancer (*P* = 0.10). The PRS effect size was higher for colorectal adenocarcinoma, the most common histological type of CRC, as compared to other histological CRC subtypes. We observed no significant differences between early-onset (age <50) and late-onset (age ≥50) CRC (effect sizes estimated using different cut-offs in Supplementary Table 5) or between local or distant spread at presentation (data available for only 60.8% of CRC cases) with the PRS.

Among individuals with positive first-degree family history (FH) of CRC with average PRS (20–80th percentile), the lifetime risk of CRC was estimated at 8.2% (95% CI 6.5–10.0%; Supplementary Fig. 6). With high PRS (> 80th percentile), the risk in individuals with positive FH increased to 11.8% (9.0–14.5%), whereas a low PRS (below the 20th percentile) compensated for the risk incurred by FH (4.7% [95% CI 2.7–6.7%]), leading to a risk level comparable to the population. Exclusion of genomic regions containing known Lynch syndrome-causing genes [37] from the PRS did not impact the effect size of neither the PRS alone nor the effect size of PRS in individuals with positive FH (Supplementary Table 3). Furthermore, the CRC PRS did not have strong associations to extracolonic cancers (Supplementary Table 6), as is often observed in cases of hereditary CRC syndromes [37–39].

**Fig. 4:**
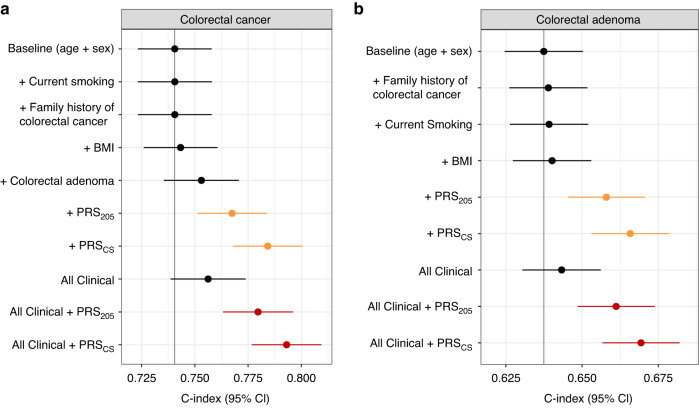
C-index for combinations of age, sex and individual risk factors. Model discrimination measured by C-index for clinical colorectal cancer (CRC) risk factors and a CRC polygenic risk score (PRS), based on a model for 10-year **a** incident CRC risk (*N* = 80,272; 721 cases) and **b** incident colorectal adenoma risk (*N* = 78,245; 1787 cases). Shown are the C-indices from Cox proportional hazards models for incident colorectal cancer (**a**) and colorectal adenoma (**b**) in FinnGen comparing the PRS with established risk factors. The baseline model included age, sex, the first ten genetic principal components of ancestry, subcohort and genotyping array as covariates. Coloured points indicate scores that include PRS; black points represent non-genetic scores. Error bars represent 95% confidence intervals for each factor with incident disease as endpoint. BMI body mass index.

Finally, we tested the PRS in the prediction of 10-year incident CRC and adenoma risk among individuals aged over 40 years old at FinnGen study recruitment without prevalent inflammatory bowel disease or primary sclerosing cholangitis. The available sample sizes and incident cases available were 80,272 individuals with 721 incident cases for CRC and 78,245 individuals with 1787 incident cases for colorectal adenoma. The median follow-up time was 10.0 years (interquartile range [IQR] 7.4–10.0) for CRC analysis and 10.0 years (IQR 7.2–10.0) for adenoma analysis. Our PRS improved discrimination for CRC over a baseline model including age and sex (increase in C-index of 0.044) more than any single non-genetic risk factor, including current smoking, FH of CRC, BMI, and personal history of colorectal adenomas (Fig. 4a). The genome-wide PRS also showed slightly better discrimination than both the PRS_205_ and all the non-genetic risk factors combined, and adding the PRS to the non-genetic risk factors improved the C-index beyond age, sex, and all non-genetic risk factors. Similar patterns were also observed for 10-year incident colorectal adenomas (Fig. 4b).

## Discussion

We developed a genome-wide PRS for CRC and evaluated its impact in the context of population-level screening for CRC. We carefully calibrated the model to respective population risk allowing estimation how different PRS categories would affect screening initiation age in existing CRC screening programmes. Our findings show that the PRS was effective in identifying individuals at high risk of CRC in general, as well as for identifying those at risk for colorectal adenomas and post-colonoscopy CRC. Furthermore, we examined the characteristics of CRCs and related precursors associated with the PRS and showed that the CRC PRS improved the prediction of 10-year risk of CRC beyond established clinical risk factors. Our study highlights the potential of using a PRS for CRC in population-level screening programmes to identify individuals at elevated risk of developing CRC and tailor screening strategies accordingly.

Our results are consistent with previous studies [12–17, 40] which have assessed CRC PRSs alone or integrated with non-genetic risk factors. In aggregate, prior analyses suggest that genome-wide PRS approaches outperform those using only a small set of genome-wide significant single-nucleotide polymorphisms, that individuals with a high CRC PRS have overall higher risk and earlier onset of CRC, and that models integrating PRS with non-genetic risk factors generally perform better than PRS or non-genetic risk factors alone. In addition, polygenic risk has been estimated to modify CRC risk for those ascertained with first-degree family history of CRC [41] or high- or moderate-impact germline variants associated with CRC risk [32]. Unlike previous studies, which have often relied on cross-sectional data or datasets that may not fully represent the background population, our study utilises a large dataset comprising 8.2% of Finns, with high-coverage nationwide cancer registry data used for rigorous calibration of our risk models. Our approach extends initial findings on how risk-based screening with CRC PRSs could be done for the population, and we conducted analyses with alternative age thresholds for screening initiation, which adds to the generalisability of our findings across different healthcare systems and European ancestry populations.

We observed large differences in lifetime CRC risks for different PRS strata, with up to 15.5% lifetime risk in men and 11.5% in women in the highest tail of the PRS, and conversely 2.2% in men and 2.1% in women in the low tail of the PRS. While our data show that the majority of individuals with an average PRS could continue following standard guidelines to begin screening, the difference in optimal screening age is more than 18 years apart at the high and low tails of the PRS, marking the potential clinical impact of incorporating PRS to risk-based screening approaches for systematic identification of at-risk young adults. Furthermore, the PRS showed independence from first-degree family history of the disease and Lynch syndrome variants while effectively stratifying risk in the presence of family history of the disease. These data hold particular clinical relevance as the rising incidence of CRC in adults younger than 50 years during the last decades [3, 42] has resulted in recent recommendations of earlier population screening in many high-income countries [2]. Importantly, the level and timing of observed risk at the population level among individuals with high PRS is comparable to that of individuals carrying risk variants in known CRC susceptibility genes [32, 43, 44] or individuals with positive first-degree family history of CRC [4], qualifying them for earlier screening under current screening guidelines [5–7, 42]. Further research is needed to determine the optimal screening modality, timing, and frequency for individuals with high PRS both alone and together with established clinical risk factors.

Surveillance recommendations after colonoscopy for prevention of CRC incorporate both index colonoscopy findings and identified risk factors [45], and high genetic risk based on family history of CRC [46] or inherited cancer syndromes [6] generally warrant more intensive surveillance than would be allocated to the general adult population [46]. For those without any identifiable risk factors or neoplastic findings in colonoscopy, a 10-year follow-up is generally considered sufficient regardless of colonoscopy indication [45]. Our results show that clinically average-risk individuals following a negative colonoscopy at the top decile of the PRS were at over twofold elevated risk for post-colonoscopy CRC compared to individuals with an average PRS. This finding, to our knowledge previously unreported in a prospective setting [47, 48], might warrant intensified surveillance of individuals with elevated PRS undergoing colonoscopy.

Our study also shows that the PRS could aid in short-term screening decisions and risk stratification. Assessed by the C-index, the PRS improved 10-year risk discrimination over age and sex when combined with non-genetic risk factors, which was not achieved by non-genetic risk factors alone, a finding consistent with recently published data from UK Biobank incorporating PRS to non-genetic risk factors [12].

The higher frequency of precursor colorectal adenomas in middle-aged individuals with higher PRS supports previous findings from case-control analyses that common genetic variants associated with CRC mediate risk at least partly through increased predisposition to precursor adenomas [49–51]. We found no evidence suggestive of differential adenoma location or cancer spread at presentation by PRS. We also did not replicate a stronger PRS association for early-onset CRC cases as compared to late-onset cases previously reported in a case-control study [14], possibly due to the limited number of early-onset CRC cases in our cohort. However, our study showed that the PRS had a larger effect size for distally located CRCs which have a predominance in early-onset disease as compared to cancers of the proximal colon, with a higher proportion of proximal cases being late-onset and post-colonoscopy CRCs [3, 23, 42].

Our large-scale cohort design leverages high-coverage nationwide registries linked to a large biobank study with careful recalibration of lifetime risk estimates. In contrast to many previous studies, we evaluated the impact of PRS on diverse clinical characteristics, such as sidedness in CRC and colorectal adenomas, and by sex. However, we were unable to precisely assess the PRS impact on precursor adenomas due to incomplete clinical information, including polyp size, type, or number, and the potential underrecording of these lesions in electronic health records. Furthermore, as our colonoscopy cohort parallels more likely a selected patient population rather than a representative screening cohort, evaluation of the PRS through prospective colonoscopy-based screening programmes are needed to further determine the strength of the association after screening colonoscopy. Our study was also limited by the lack of information on quality indicators of colonoscopy based on registry data. However, Finland has established national quality-assurance guidelines for colonoscopies [52]. Our findings on the performance of PRS are generalisable across European ancestry, but similar evaluations are needed for diverse ethnic groups, considering the low transferability of PRSs across ancestries [53].

In conclusion, we developed a genome-wide polygenic risk score for CRC and demonstrated its effectiveness in identifying individuals at high risk of CRC, related precursors, and post-colonoscopy CRC. Our findings support the use of a CRC PRS for risk stratification in CRC detection and prevention, showing also benefit when added to non-genetic clinical risk factors. Further research is needed to determine how to optimally integrate a CRC PRS into prospective risk-based screening, including evaluation of cost-effectiveness.

### Supplementary information

Supplementary Information

Supplementary Data 1

Supplementary Data 2

## Data Availability

The Finnish biobank data can be accessed through the Fingenious® services (web link: https://site.fingenious.fi/en/, email: contact@finbb.fi). Linkage disequilibrium reference panels constructed using the 1000 Genomes Project [29] Phase 3 samples can be downloaded at https://github.com/getian107/PRScs. The weights for our genome-wide PRS built with PRS-CS are available at PGS Catalog [54] (pgs-info@ebi.ac.uk) at https://www.pgscatalog.org/score/PGS003979/. The weights for the PRS_205_ are available at https://www.pgscatalog.org/score/PGS003850/.

## Code availability

The full genotyping and imputation protocol for FinnGen is described at 10.17504/protocols.io.xbgfijw. The PRS-CS pipeline in FinnGen is described at https://github.com/FINNGEN/CS-PRS-pipeline. All software packages and programmes used to perform these analyses are freely available, and can be found within the manuscript and the Supplementary Information. The code used for these analyses are available from the corresponding author upon reasonable request.
