# Supplementary material for: Genome-wide polygenic risk scores for colorectal cancer have implications for risk-based screening
Source: Br J Cancer. 2024 Jan 3;130(4):651–9. doi: 10.1038/s41416-023-02536-z (PMC10876651; doi:10.1038/s41416-023-02536-z)
Supplement: Supplementary file 1 — Supplementary Information [file 41416_2023_2536_MOESM1_ESM.docx]

**SUPPLEMENTARY INFORMATION**

**Supplementary Table 1.** Subcohorts and biobanks in FinnGen Data Freeze 11.

| **Cohort / Biobank** | **N individuals** |
| --- | --- |
| ARCTIC BIOBANK NFBC1966 | 5792 |
| ARCTIC BIOBANK NFBC1986 | 5318 |
| ARCTIC BIOBANK OULU1935 | 432 |
| ARCTIC BIOBANK OULU1945 | 591 |
| AURIA BIOBANK* | 53,523 |
| BIOBANK OF CENTRAL FINLAND* | 12,434 |
| BIOBANK OF EASTERN FINLAND* | 22,430 |
| BLOOD SERVICE BIOBANK | 51,012 |
| BOREALIS BIOBANK* | 14,330 |
| HELSINKI BIOBANK* | 98,202 |
| TAMPERE BIOBANK* | 36,675 |
| TERVEYSTALO BIOBANK | 20,607 |
| THL BIOBANK ATBC | 18,243 |
| THL BIOBANK BOTNIA | 10,526 |
| THL BIOBANK COROGENE | 4416 |
| THL BIOBANK FINHEALTH 2017 | 5752 |
| THL BIOBANK FINHIT | 4349 |
| THL BIOBANK FinIPF | 275 |
| THL BIOBANK FINRISK 1992 | 4909 |
| THL BIOBANK FINRISK 1997 | 6970 |
| THL BIOBANK FINRISK 2002 | 6905 |
| THL BIOBANK FINRISK 2007 | 5099 |
| THL BIOBANK FINRISK 2012 | 5216 |
| THL BIOBANK FUSION | 4276 |
| THL BIOBANK GENERISK | 6817 |
| THL BIOBANK HEALTH 2000 | 6388 |
| THL BIOBANK HEALTH 2011 | 707 |
| THL BIOBANK HHS | 3334 |
| THL BIOBANK KUUSAMO | 229 |
| THL BIOBANK MIGRAINE | 8240 |
| THL BIOBANK SUPER | 8482 |
| THL BIOBANK T1D | 10,110 |
| THL BIOBANK TWINS | 11,144 |

*Hospital-based biobanks.

**Supplementary Table 2.** Sensitivity analysis of the impact of potentially overlapping samples between GWAS used for generating the PRS and FinnGen.

|  | **OR per 1 SD (95% CI) change in PRS** |
| --- | --- |
|  |  |
| FinnGen (N = 453,733, 8801 CRC cases) | 1.63 (1.60–1.67) |
| FinnGen, overlapping samples excluded*  (N = 429,858, 8016 CRC cases) | 1.62 (1.59–1.66) |

*Potentially overlapping samples were excluded based on genotyping array information. The estimates are from logistic regression models adjusted for year of birth, sex, the ten first principal components of ancestry, batch, and genotyping array. The number of CRC cases was 8801 in the full FinnGen dataset and 8701 in the sensitivity analysis.

**Supplementary Table 3.** Effect size of a PRS containing 205 variants, compared to genome-wide PRSs with and without including regions containing Lynch syndrome variants among all individuals in the full FinnGen dataset and in individuals with first-degree family history of CRC.

| **Statistic** | | **PRS_205_^1^** | | **PRS_CS_ (main PRS studied)** | **PRS_CS_ with LS variants excluded^2^** |
| --- | --- | --- | --- | --- | --- |
| Number of variants | | 183 / 205 | | 1,088,133 | 1,082,582 |
| **All individuals (N = 453,733)** | | | | | |
| OR per SD (95% CI) | | 1.51 (1.48–1.55) | | 1.63 (1.60–1.67) | 1.62 (1.59–1.66) |
| AUC (95% CI) | | 0.788 (0.784–0.792) | | 0.795 (0.791–0.799) | 0.795 (0.791–0.799) |
| AUC* (95% CI) | | 0.721 (0.716–0.726) | | 0.732 (0.727–0.737) | 0.732 (0.727–0.736) |
| PR-AUC^†^ | | 0.063 | | 0.068 | 0.067 |
| AIC^‡^ | | 77366.5 | | 76818.5 | 76853.4 |
| **Individuals with FH for CRC (N = 9,185)** | | | | | |
| OR per SD (95% CI) |  | | 1.39 (1.24–1.55) | 1.47 (1.32–1.65) | 1.50 (1.34–1.67) |
| AUC (95% CI) | | 0.786 (0.765–0.807) | | 0.789 (0.768–0.809) | 0.791 (0.771–0.811) |
| AUC* (95% CI) | | 0.772 (0.745–0.794) | | 0.773 (0.757–0.795) | 0.775 (0.754–0.797) |
| PR-AUC^†^ | | 0.15 | | 0.15 | 0.15 |
| AIC^‡^ | | 2858.7 | | 2846.0 | 2842.2 |
|  | |  | |  |  |

Effect sizes are shown separately for all individuals and for those with positive first-degree family history (FH). Shown for each of three PRSs are number of variants and statistical indicators in FinnGen (N = 453,733) with 8,801 cases of CRC in the full FinnGen dataset and separately in individuals with FH for CRC (N = 9,185 with 342 cases of CRC). The odds ratio (OR) and area under the receiver operating characteristic curve (AUC) estimates are from logistic regression models adjusted for year of birth, sex, and additionally ten first principal components of ancestry, batch, and genotyping array in FinnGen. ^1^183 out of 205 variants available and polymorphic in FinnGen. ^2^To create a PRS independent of Lynch Syndrome (LS) variants, we excluded 60,656 single nucleotide polymorphisms within ±2 megabases of the *MLH1*, *MSH2*, *MSH6* and *PMS2* genes from the discovery GWAS summary statistic which we then used to generate the PRS. FH, first-degree family history, *PRS_205_* PRS with 205 genetic variants^1^ with 183 variants polymorphic in FinnGen, *PRS_CS_* PRS calculated with the software PRS-CS. PR-AUC, area under the precision recall curve. AIC, Akaike information criterion. *AUC estimates from logistic regression models adjusted for ten first principal components of ancestry, batch, and genotyping array in FinnGen. †Measures the fraction of true positives among positive predictions, higher values indicate greater discrimination. ‡Measures prediction error, smaller values indicate better model fit.

**Supplementary Table 4.** Baseline characteristics of FinnGen Data Freeze 11 individuals.

|  | **Men** | **Women** |
| --- | --- | --- |
| **N** | 199,115 | 254,618 |
| **Baseline Age, mean ± SD** | 55.1±17.5 | 51.2±18.1 |
| **Prevalent CRC** | |  |
| Proximal colon, N (%) | 1027 (0.52) | 1098 (0.43) |
| Distal colon, N (%) | 530 (0.27) | 389 (0.15) |
| Unspecified colon, N (%) | 172 (0.086) | 170 (0.067) |
| Rectum, N (%) | 1226 (0.62) | 858 (0.34) |
| **Incident CRC** | |  |
| Proximal colon, N (%) | 783 (0.39) | 539 (0.21) |
| Distal colon, N (%) | 499 (0.25) | 178 (0.070) |
| Unspecified colon, N (%) | 163 (0.082) | 135 (0.053) |
| Rectum, N (%) | 715 (0.36) | 319 (0.13) |
| **Prevalent Adenoma** | |  |
| Proximal colon, N (%) | 1886 (0.95) | 1847 (0.73) |
| Distal colon, N (%) | 1976 (0.99) | 1849 (0.73) |
| Unspecified colon, N (%) | 2842 (1.43) | 2424 (0.95) |
| Rectum, N (%) | 1262 (0.63) | 1311 (0.51) |
| **Incident Adenoma** | |  |
| Proximal colon, N (%) | 3608 (1.81) | 4436 (1.74) |
| Distal colon, N (%) | 657 (0.33) | 490 (0.19) |
| Unspecified colon, N (%) | 1162 (0.58) | 738 (0.29) |
| Rectum, N (%) | 934 (0.47) | 778 (0.31) |
| **Age at onset** | |  |
| Early-onset CRC (<50y), N (%) | 322 (0.16) | 485 (0.19) |
| Late-onset CRC (≥50y), N (%) | 4793 (2.41) | 3201 (1.26) |
| **First-degree Family History of CRC**, N (%) | 3955 (1.99) | 5230 (2.05) |
| **Prevalent IBD**, N (%) | 5185 (2.60) | 6070 (2.38) |
| **BMI*, kg/m^2^, mean ± SD** | 27.3±4.93 | 27.3±6.04 |
| **Current Smoking***, N (%) | 46494 (23.4) | 27717 (10.9) |

Prevalent cases were defined as cases that occurred before FinnGen study entry. *Individuals with missingness excluded (47,872 men and 84,189 women for BMI, 68,723 men and 107,914 women for Current Smoking). CRC, colorectal cancer. IBD, inflammatory bowel disease. BMI, body mass index.

**Supplementary Table 5.** CRC PRS odds ratios (OR) from logistic regression using different cut-offs for age at CRC diagnosis in FinnGen (total sample size N = 453,733, number of CRC cases 8,801).

| **Age at CRC diagnosis** | **N cases/controls** | **OR (95% CI) per SD of PRS** | ***p*** |
| --- | --- | --- | --- |
| < 45 years | 479/444,932 | 1.38 (1.27–1.52) | 1.60 × 10^-12^ |
| ≥ 45 years | 8322/444,932 | 1.63 (1.59–1.67) | < 1.00 × 10^-300^ |
|  |  |  |  |
| < 50 years | 807/444,932 | 1.51 (1.41–1.62) | 1.75 × 10^-31^ |
| ≥ 50 years | 7994/444,932 | 1.63 (1.60–1.67) | < 1.00 × 10^-300^ |
|  |  |  |  |
| < 55 years | 1359/444,932 | 1.59 (1.51–1.68) | 9.07 × 10^-65^ |
| ≥ 55 years | 7442/444,932 | 1.63 (1.59–1.66) | < 1.00 × 10^-300^ |
|  |  |  |  |
| < 60 years | 2177/444,932 | 1.65 (1.58–1.73) | 3.46 × 10^-119^ |
| ≥ 60 years | 6624/444,932 | 1.61 (1.57–1.64) | < 1.00 × 10^-300^ |
|  |  |  |  |
| < 65 years | 3328/444,932 | 1.67 (1.61–1.73) | 8.56 × 10^-186^ |
| ≥ 65 years | 5473/444,932 | 1.59 (1.55–1.64) | 4.13 × 10^-249^ |

The control groups in logistic regression analyses were defined as healthy control individuals without history of CRC. The models were adjusted for age, birth year, genotyping array, subcohort, and the first ten genetic principal components of ancestry.

**Supplementary Table 6.** CRC PRS hazard ratios (HR) from Cox regression for extracolonic cancers in FinnGen (total sample size N = 453,733).

| **Extracolonic Cancer** | **N events** | **HR (95% CI) per SD change in CRC PRS** | ***p*** |
| --- | --- | --- | --- |
| Any cancer excl. colorectal cancer | 83,856 | 1.03 (1.02–1.04) | 1.03 × 10^-7^ |
| Gastric | 1519 | 1.04 (0.99–1.09) | 0.13 |
| Small intestine | 608 | 1.10 (1.01–1.19) | 0.024 |
| Pancreatic or biliary | 2816 | 1.07 (1.03–1.11) | 0.00031 |
| Breast | 19,731 | 1.02 (1.01–1.04) | 0.003 |
| Ovarian | 1350 | 1.00 (0.95–1.06) | 0.94 |
| Endometrial | 1955 | 1.04 (0.99–1.08) | 0.11 |
| Urinary tract | 2990 | 1.00 (0.97–1.04) | 0.86 |
| Skin | 23,901 | 1.01 (1.00–1.02) | 0.13 |
| Central nervous system | 1007 | 0.95 (0.90–1.01) | 0.13 |

The models were adjusted for ten first principal components of ancestry, batch, and genotyping array in FinnGen. The cancers presented were selected to include the most common extracolonic cancers in patients with Lynch Syndrome^2-4^. The phenotype definitions are shown in Supplementary Data 1.

**Supplementary Figure 1.** Goodness-of-fit of the colorectal cancer polygenic risk scores (PRS) for colorectal cancer. The x-axis and y-axis represent the expected and observed proportions of cases, respectively. The PRS is divided into 20 bins of equal size.


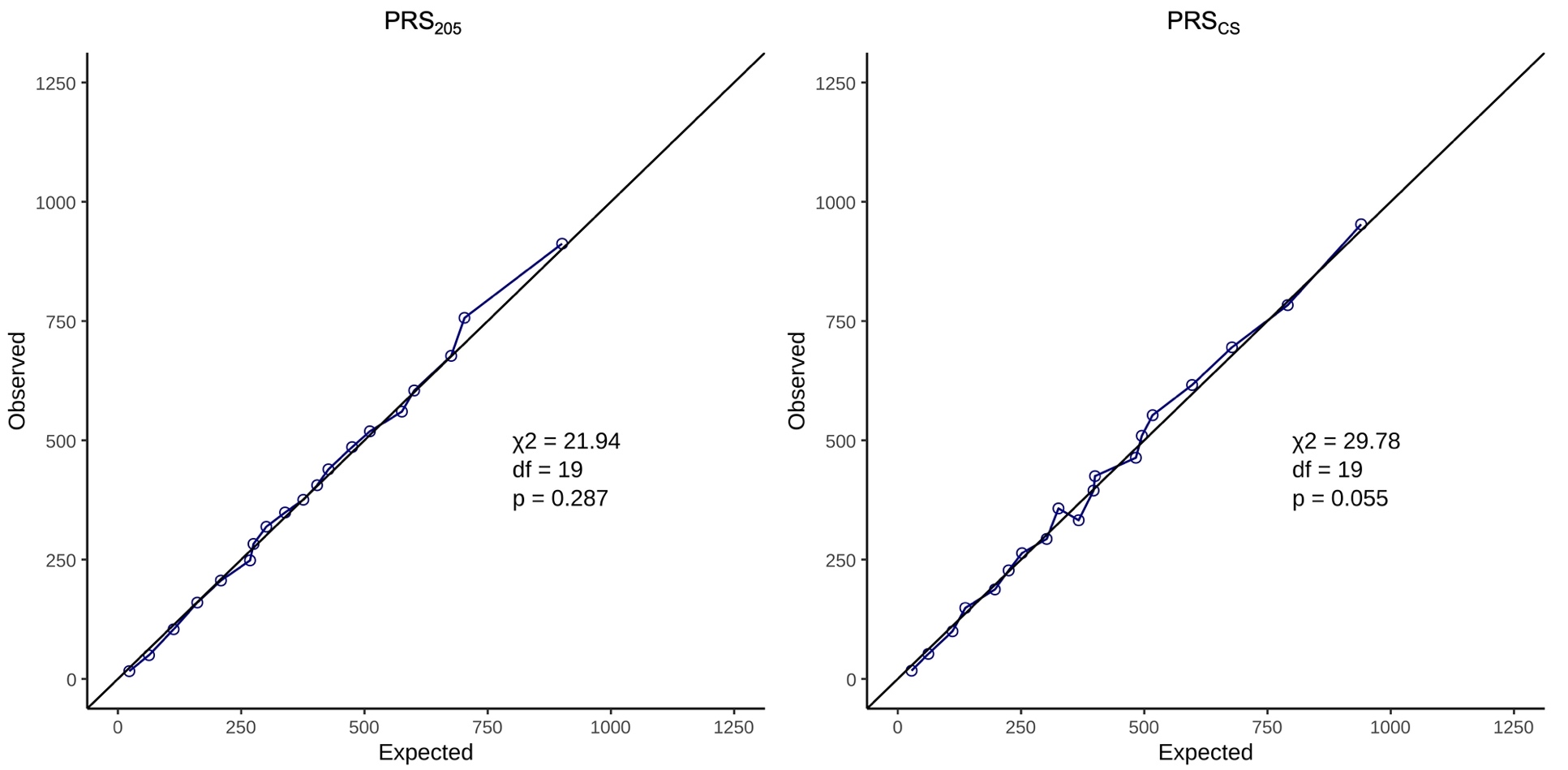


PRS_205_ = a PRS with 205 genetic variants^1^, with 183 variants available in FinnGen. PRS_CS_ = a PRS built with the software PRS-CS. Goodness-of-fit for the Cox proportional hazards model was assessed with the R package survMisc using the function *gof*, which calculates the expected incidence in each of the 20 bins, based on an object from the function *coxph* (R package survival). In the regression analysis, we used a continuous PRS to estimate effects per standard deviation, and used age as the time scale, with batch, subcohorts and the first ten principal components of ancestry as covariates. The shown summary statistics from likelihood ratio tests indicate model fit.

**Supplementary Figure 2.** Age- and sex-specific lifetime risks of CRC in Finland.


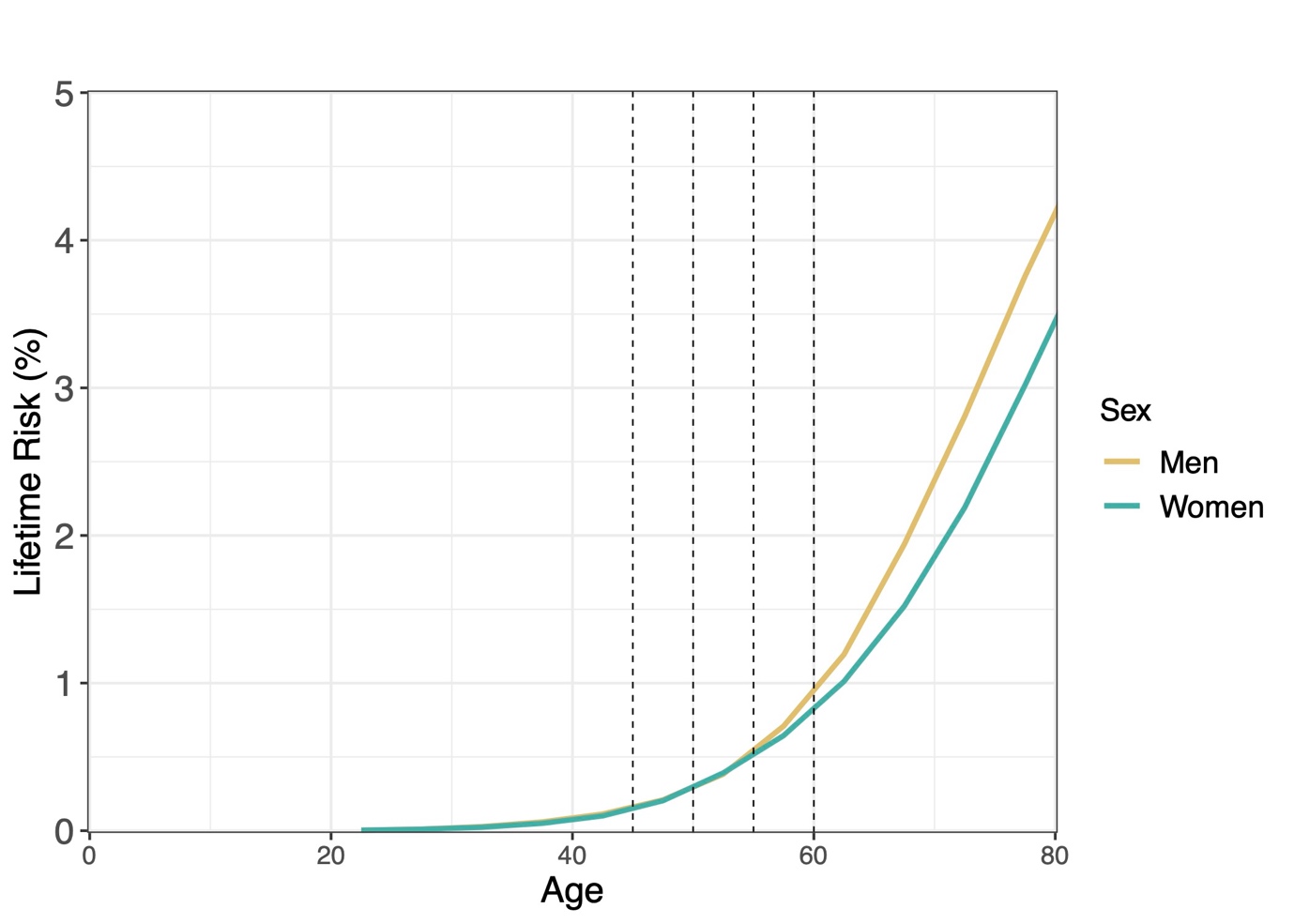


We transformed our age-specific calibrated estimates to a continuous scale by using the age group medians and used linear interpolation to determine ages when the cumulative incidence reached thresholds for screening initiation. These estimated incidences in the Finnish population were utilized in our PRS calibration approach (see Methods). The vertical lines depict ages (45, 50, 55, 60 years of age) when screening is commonly initiated. We used linear interpolation to determine the cumulative incidences at these ages which were used in Table 1.

**Supplementary Figure 3.** Lifetime risk of CRC and recommended CRC screening initiation age according to polygenic risk score (PRS) deciles in men and women.


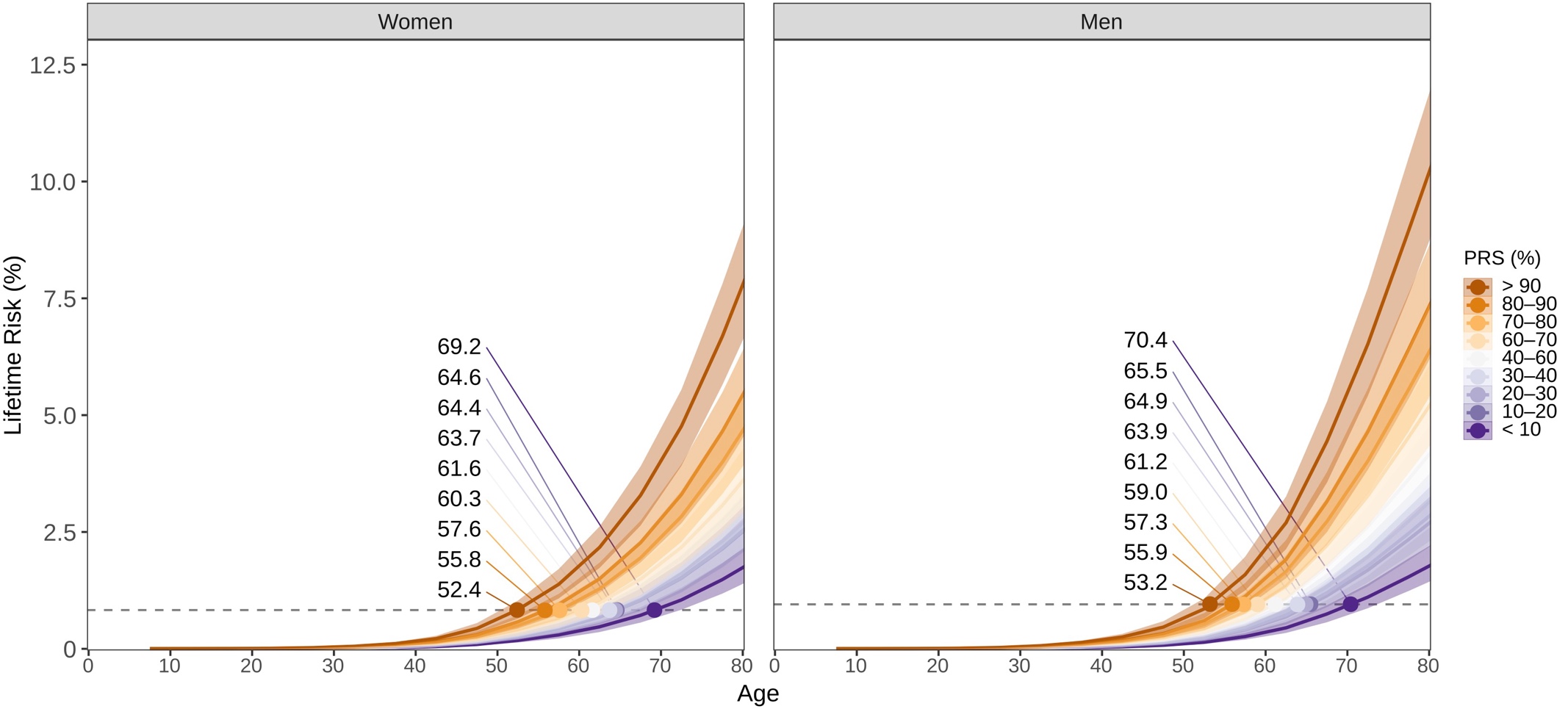


The dashed line indicates the cumulative incidence for the population average for 60-year-old women (0.83%) and men (0.95%) in the Finnish population. The colored points with respective numbers show the age at which each polygenic risk score (PRS) category reaches the same cumulative incidence as the average population. The shaded bands represent 95% confidence intervals.

**Supplementary Figure 4.** Cumulative risk of colorectal adenoma among FinnGen individuals who have undergone colonoscopy (N = 86,708, 21,012 adenoma cases by age 80).

**
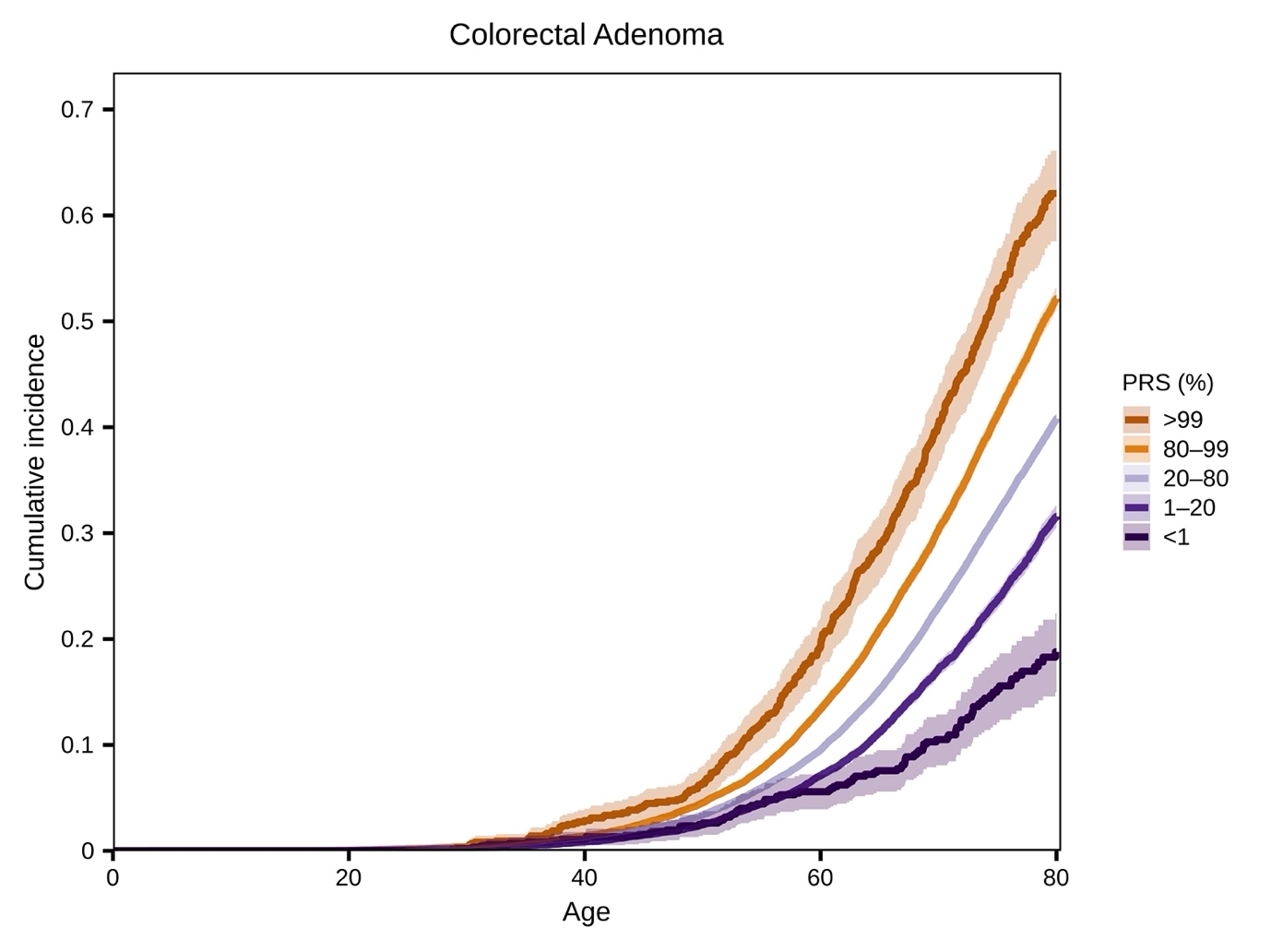
**

The survival curves show cumulative risk for colorectal adenoma for individuals who had PRS in one of the following categories: less than 1%, 1–20%, 20 to 80%, 80–99% and above 99%. The shaded bands represent 95% confidence intervals.

**Supplementary Figure 5.** Distributions of polygenic risk score and age at index colonoscopy by post-colonoscopy CRC status (N = 48,638 individuals with 214 cases).

Shown are distributions for PRS and age at index colonoscopy in post-colonoscopy CRC analysis. Using adjusted Cox regression, the continuous PRS hazard ratio per one SD change in the PRS was 1.76 (95% CI 1.54–2.01, *P* = 2.36 × 10^-16^). The hazard ratio for each 1-unit increase in age at index colonoscopy was 1.059 (1.044–1.074, *P* = 2.76 × 10^-15^). The model included PRS, age at index colonoscopy, the first ten genetic principal components of ancestry, genotyping batch and subcohort as covariates. PRS_CRC_, genome-wide PRS built with PRS-CS.

**Supplementary Figure 6.** The impact of polygenic risk on disease risk in individuals with positive first-degree family history for CRC (N = 9,185 individuals with FH).


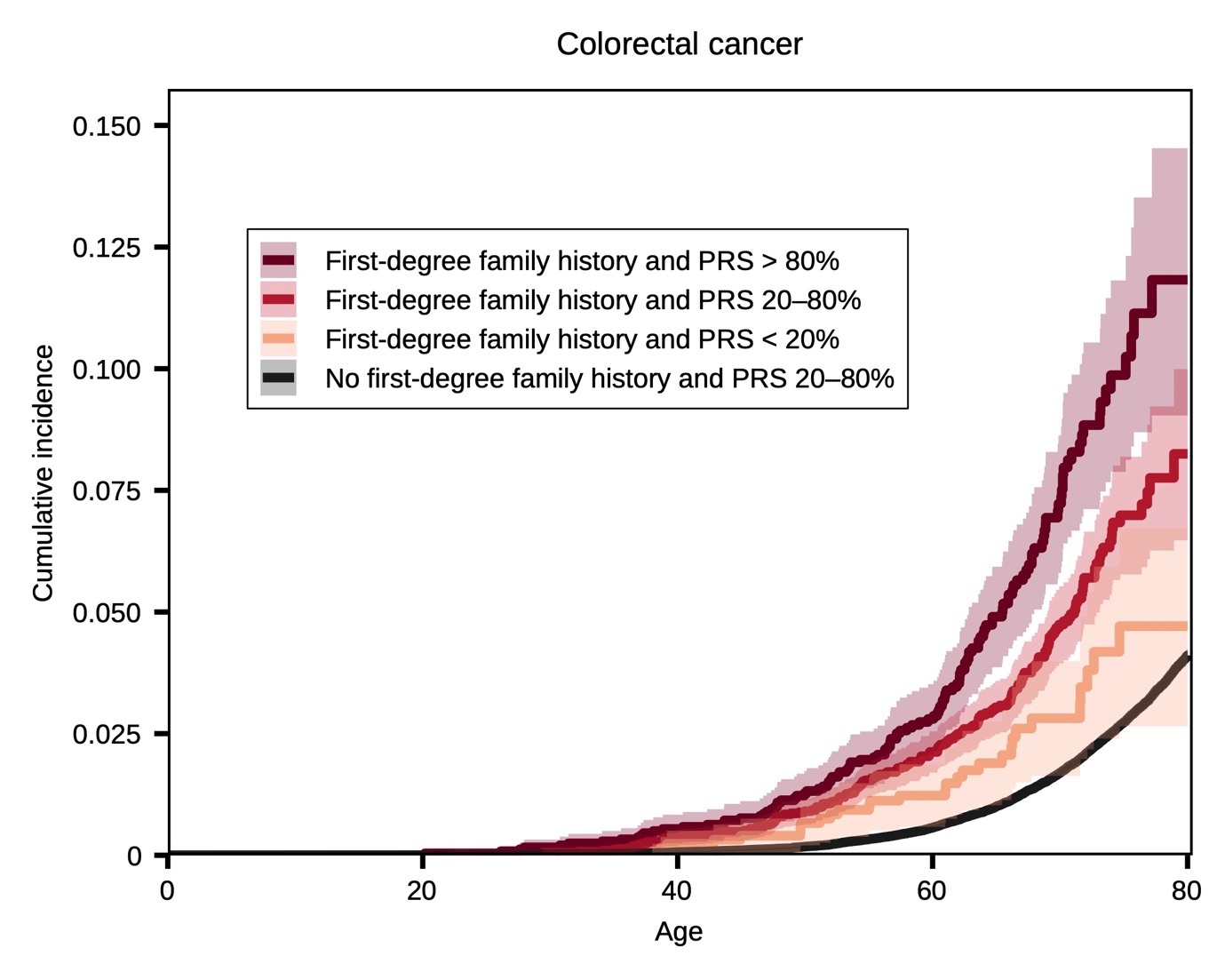


The survival curves show cumulative incidences for individuals with positive first-degree family (FH), stratified by polygenic risk score (PRS). High PRS was defined as top quintile of the PRS distribution and low PRS as the bottom quintile of the PRS distribution. Total N = 9,185 individuals with FH with 337 cases of CRC by age 80. The number of individuals without presence of first-degree family history with average PRS (20–80%) was 266,837 with 3894 cases of CRC by age 80.

**SUPPLEMENTARY METHODS**

**Study design and participant characteristics**

The FinnGen study, detailed at (<https://www.finngen.fi/en>) and in Kurki et al.^5^, is an ongoing research project utilizing samples sourced from a network of Finnish biobanks and electronic healthcare records from national health registries. The FinnGen project contains phenotype and genotype data for approximately 500,000 individuals, and sample collection and data releases started in 2017. The main phase of sample collection ends in 2023. The FinnGen study includes both legacy samples (estimated approximately 200,000 individuals) originating from previous research cohorts, including specialized disease-based cohorts, which are stored in Finnish biobanks. Additionally, prospective samples (predicted approximately 300,000 individuals) are collected by various biobanks throughout Finland. These prospective samples from six regional hospital biobanks include an array of patients recruited by specialized healthcare. Furthermore, participants enlisted through the Blood Service Biobank and private healthcare biobanks contribute to the cohort by introducing healthier individuals. In the current study, we used samples from 453,733 biobank participants. We used first recorded events for each phenotype in our analyses captured through diagnosis, reimbursement, and procedure coding in high-coverage registry data (Supplementary Data 1). The subcohorts and biobanks included in FinnGen Data Freeze 11 are shown in Supplementary Table 1. The study design and participant characteristics of the discovery genome-wide association study meta-analysis by Fernandez-Rozadilla et al. used to construct our PRSs, along with other relevant details, have been previously published^1^.

**Genotyping, imputation, and quality control**

FinnGen samples were genotyped with Illumina (Illumina Inc., San Diego, CA, USA) and Affymetrix arrays (Thermo Fisher Scientific, Santa Clara, CA, USA). The genotyping of prospective samples is performed using the FinnGen ThermoFisher Axiom custom array (V2) containing 736,145 probesets for 655,973 genetic markers. In addition to approximately 500,000 core GWAS markers, the array contains 116,402 coding variants enriched in Finland, >10,000 specific markers for the HLA/KIR region and approximately 15,000 ClinVar and 4,600 pharmacogenomic variants, and further 57,000 selected markers that were of special interest to FinnGen consortium partners. Genotype calls were made with the GenCall or zCall (for Illumina) and the AxiomGT1 algorithm (for Affymetrix).

Genotype imputation in FinnGen Data Freeze 11 was performed with Beagle 4.1 by using the Sequencing Initiative Suomi (SISu) v4 population-specific reference panel developed from high-quality data for 8,554 high-coverage (25–30×) whole-genome sequences in Finns. The variant call set for the imputation reference panel was produced with the GATK HaplotypeCaller algorithm following GATK best practices for variant calling, and genotype-, sample, and variant-wise quality control (QC) were applied by an iterative manner on the high-coverage whole-genome sequencing data using the Hail framework v0.1 (<https://github.com/hail-is/hail>). Detailed sample QC included removing samples where genetic sex did not match provided sex from registries (female: ƒ < 0.4, male: ƒ > 0.7), samples with excess heterozygosity (> 3 standard deviations from the mean) in common variants (allele frequency > 0.05) per batch and samples with excess relatedness to other samples ($\hat{\pi}$> 0.1). Variant QC included mapping variants to reference genome (including left alignment, minimization of allele representation and variant annotation) and removal of variants with alleles not represented with [ATGC]. For imputed variants, variants were removed if they were not in the imputation reference panel or if the variant allele frequency was < 0.001 in the imputation panel or if the allele frequency differed significantly from the imputation panel (*P* < 5 × 10^-8^) adjusted for the first 10 principal components. Furthermore, variants were removed from all batches if the variant Hardy–Weinberg equilibrium p-value was < 1 × 10^−10^ across all batches (except if there were fewer than expected homozygotes for variants with MAF < 0.015) or if more than 15% of the batches had variant missingness > 4% or the variant was non-PASS in more than 30% of the batches. Lastly, variants were removed within a batch if they had high missingness (>2%), had low Hardy–Weinberg equilibrium p value (< 1 × 10^−6^) or were non-PASS. BCFtools 1.7 and 1.9 and PLINK v2.00a2.3LM were used for data and variant handling and PRS calculation. Cromwell 61 was used for workflow handling. Array data pre-phasing was carried out with Eagle 2.4.1 with default parameters, except that the number of conditioning haplotypes was set to 20,000. The reference assembly used was GRCh38/hg38. The total number of samples passing genotyping QC was 473,681. Further details of FinnGen samples have been previously described^5^.

**Population structure**

For population structure analysis, the 473,681 samples that passed genotyping QC were combined with 2,504 phase 3 reference samples from the 1000 Genomes Project and principal component analysis was used to identify FinnGen participants who had non-Finnish genetic ancestry. Most participants had broadly genetically inferred Finnish ancestry (17,155 out of 473,681 [3.62%] outliers were removed). A total of 19,784 duplicates and monozygotic twins (one from each pair randomly removed) and genetic population outliers were removed resulting in a set of approximately unrelated individuals for which the relation between any pair is third degree or higher. In total, the data contained 277,053 independent individuals, which were used to compute the principal component analysis, and data for the 176,844 related individuals were projected onto these principal components. Out of these total remaining 453,897 genotyped samples, 453,733 (56.1% females) had phenotype data and genetic sex matching to sex provided by registries and were included in all analyses, with the baseline participant characteristics and prevalent and incident case status shown in Supplementary Table 4. Further details on population structure in FinnGen have been previously reported^5^.

**Disease endpoints and risk factor definitions**

For incident disease and family history analyses, we identified risk factors from either baseline measurements or questionnaire data collected by biobanks (body mass index [kg/m^2^] and smoking status) or through national registries. First-degree family history of colorectal cancer (CRC) was defined based on inferred pairs of first-degree relatives (inference based on 57 K unlinked variants in KING v2.2.431; kinship coefficient range 0.177 to 0.354), or from parental causes of death data that were linked through the causes of death registry for the full FinnGen data (available from 1971 to 2020) or by using ICD-10 code Z80.0. First-degree family history for CRC was set to start from FinnGen study baseline in all analyses.

For post-colonoscopy CRC ascertainment, we identified 86,708 FinnGen individuals who had undergone colonoscopy between January 1, 1986 (start of registry coverage of procedures in Finland), and 31 December 2021. Of these individuals, 76,113 were at least 40 years of age at the date of the examination. Colonoscopies were identified from hospital discharge registries using NOMESCO Classification of Surgical Procedures, Finnish Hospital League surgical procedure codes and SPAT procedure codes (Supplementary Data 1). We selected the first available colonoscopy as the index examination and only included individuals who were at average clinical risk before the index examination, which we defined as not having diagnosis of inflammatory bowel disease (N = 5,143 individuals with inflammatory bowel disease) or primary sclerosing cholangitis (N = 399), previous diagnosis of CRC or diagnosis of CRC within six months of the index colonoscopy (N = 5,817), diagnosis of colorectal adenoma within three months before or after the date of the index colonoscopy in electronic health records (N = 15,941), previous proctectomy, colectomy or proctocolectomy, i.e., bowel resection affecting the rectum and/or colon (N = 3,705) or first-degree family history of CRC (N = 2,371). The final sample size for the post-colonoscopy CRC analyses comprised 48,638 individuals. These individuals were followed using the Finnish Cancer Registry and death registry data for the occurrence of post-colonoscopy CRC during 6 months to 10 years after the index colonoscopy. The follow-up ended at the age of record of post-colonoscopy CRC, age at death for a cause other than CRC, or age at last record available in the registries or electronic health records on 2 November 2022 or ten years after the index colonoscopy, whichever came first.

In incident CRC and colorectal adenoma analyses, we selected individuals aged at least 40 years at recruitment who were recruited before 2017 (N = 107,955). Of these individuals, we excluded those with no recorded body mass index or smoking status (N = 25,828), those with prevalent CRC (N = 631), inflammatory bowel disease (N = 1,359) and primary sclerosing cholangitis (N = 649). In incident adenoma analysis, we further excluded those with prevalent colorectal adenoma (N = 2,027).

**Supplementary statistical analysis**

When estimating population-calibrated lifetime risks of CRC in the Finnish population, we used a bootstrapping approach to calculate 95% confidence intervals by randomly sampling from the distribution of the error for each estimated parameter for both baseline and Cox models and recalculated the cumulative incidence 1000 times, using the 2.5^th^ and 97.5^th^ percentiles of these results. We transformed our age-specific calibrated estimates to a continuous scale by using the age group medians and used linear interpolation to determine ages when the cumulative incidence reached thresholds for screening initiation. The thresholds were based on the average cumulative risks of CRC for both men and women in the Finnish population in the year 2019 (Supplementary Figure 2) at ages 45 (0.16% for men; 0.15% for women), 50 (0.30%; 0.30%), 55 (0.55%; 0.52%) and 60 (0.95%; 0.83%).

Association between PRS and disease characteristics (cancer and adenoma anatomical location, early- and late-onset case status, tumor histology and tumor spread) were analysed separately by sex using logistic regression, with models adjusted for age, birth year, genotyping array, subcohort, and the first ten genetic principal components of ancestry. Interaction P values were estimated by including an interaction term with sex and the PRS on continuous scale (scaled to zero mean and unit variance). The control groups in logistic regression analyses were defined as healthy control individuals without history of CRC (in CRC analysis) and colorectal adenomas (in adenoma analysis).

In incident disease analyses, we used Cox model with standardized PRS on the continuous scale and the follow-up started at FinnGen study recruitment and we restricted the follow-up to a maximum of ten years after baseline and used the first ten genetic principal components of ancestry, genotype batch, subcohort, and clinical risk factors as covariates. We evaluated the incremental value of each individual non-genetic risk factor and the PRS when added on top of with age and sex and the C-index of all non-genetic risk factors combined with and without the PRS. We estimated model discrimination with the C-index, obtaining 95% CIs with normal approximation.

REFERENCES

1. Fernandez-Rozadilla C, Timofeeva M, Chen Z, Law P, Thomas M, Schmit S, et al. Deciphering colorectal cancer genetics through multi-omic analysis of 100,204 cases and 154,587 controls of European and east Asian ancestries. Nature Genetics. 2023;55(1):89-99.

2. Dominguez-Valentin M, Sampson JR, Seppälä TT, Ten Broeke SW, Plazzer JP, Nakken S, et al. Cancer risks by gene, age, and gender in 6350 carriers of pathogenic mismatch repair variants: findings from the Prospective Lynch Syndrome Database. Genet Med. 2020;22(1):15-25.

3. Lynch HT, De La Chapelle A. Hereditary Colorectal Cancer. New England Journal of Medicine. 2003;348(10):919-32.

4. Anaya DA, Chang GJ, Rodriguez-Bigas MA. Extracolonic manifestations of hereditary colorectal cancer syndromes. Clin Colon Rectal Surg. 2008;21(4):263-72.

5. Kurki MI, Karjalainen J, Palta P, Sipilä TP, Kristiansson K, Donner KM, et al. FinnGen provides genetic insights from a well-phenotyped isolated population. Nature. 2023;613(7944):508-18.

| **FinnGen** |  |  |  |  |
| --- | --- | --- | --- | --- |
| **Full Name** | **Affiliation** | **E-mail** | **Role 1** | **Role 2** |
| Aarno Palotie | Institute for Molecular Medicine Finland (FIMM), HiLIFE, University of Helsinki, Helsinki, Finland; Broad Institute of MIT and Harvard; Massachusetts General Hospital | aarno.palotie@helsinki.fi | **Steering Committee** | **Steering Committee** |
| Mark Daly | Institute for Molecular Medicine Finland (FIMM), HiLIFE, University of Helsinki, Helsinki, Finland; Broad Institute of MIT and Harvard; Massachusetts General Hospital | mark.daly@helsinki.fi | **Steering Committee** | **Steering Committee** |
| Bridget Riley-Gills | Abbvie, Chicago, IL, United States | bridget.rileygillis@abbvie.com | **Steering Committee** | **Pharmaceutical companies** |
| Howard Jacob | Abbvie, Chicago, IL, United States | howard.jacob@abbvie.com | **Steering Committee** | **Pharmaceutical companies** |
| Dirk Paul | Astra Zeneca, Cambridge, United Kingdom | dirk.paul@astrazeneca.com | **Steering Committee** | **Pharmaceutical companies** |
| Slavé Petrovski | Astra Zeneca, Cambridge, United Kingdom | slav.petrovski@astrazeneca.com | **Steering Committee** | **Pharmaceutical companies** |
| Heiko Runz | Biogen, Cambridge, MA, United States | heiko.runz@biogen.com | **Steering Committee** | **Pharmaceutical companies** |
| Sally John | Biogen, Cambridge, MA, United States | sally.john@biogen.com | **Steering Committee** | **Pharmaceutical companies** |
| George Okafo | Boehringer Ingelheim, Ingelheim am Rhein, Germany | george.okafo@boehringer-ingelheim.com | **Steering Committee** | **Pharmaceutical companies** |
| Robert Plenge | Bristol Myers Squibb, New York, NY, United States | robert.plenge@bms.com | **Steering Committee** | **Pharmaceutical companies** |
| Joseph Maranville | Bristol Myers Squibb, New York, NY, United States | joseph.maranville@bms.com | **Steering Committee** | **Pharmaceutical companies** |
| Mark McCarthy | Genentech, San Francisco, CA, United States | mccarthy.mark@gene.com | **Steering Committee** | **Pharmaceutical companies** |
| Margaret G. Ehm | GlaxoSmithKline, Collegeville, PA, United States | meg.g.ehm@gsk.com | **Steering Committee** | **Pharmaceutical companies** |
| Kirsi Auro | GlaxoSmithKline, Espoo, Finland | kirsi.m.auro@gsk.com | **Steering Committee** | **Pharmaceutical companies** |
| Simonne Longerich | Merck, Kenilworth, NJ, United States | simonne.longerich@merck.com | **Steering Committee** | **Pharmaceutical companies** |
| Anders Mälarstig | Pfizer, New York, NY, United States | anders.malarstig@pfizer.com | **Steering Committee** | **Pharmaceutical companies** |
| Katherine Klinger | Translational Sciences, Sanofi R&D, Framingham, MA, USA | katherine.klinger@sanofi.com | **Steering Committee** | **Pharmaceutical companies** |
| Clement Chatelain | Translational Sciences, Sanofi R&D, Framingham, MA, USA | clement.chatelain@sanofi.com | **Steering Committee** | **Pharmaceutical companies** |
| Matthias Gossel | Translational Sciences, Sanofi R&D, Framingham, MA, USA | matthias.gossel@sanofi.com | **Steering Committee** | **Pharmaceutical companies** |
| Karol Estrada | Maze Therapeutics, San Francisco, CA, United States | kestrada@mazetx.com | **Steering Committee** | **Pharmaceutical companies** |
| Robert Graham | Maze Therapeutics, San Francisco, CA, United States | rgraham@mazetx.com | **Steering Committee** | **Pharmaceutical companies** |
| Dawn Waterworth | Janssen Research & Development, LLC, Spring House, PA, United States | dwaterwo@its.jnj.com | **Steering Committee** | **Pharmaceutical companies** |
| Chris O´Donnell | Novartis Institutes for BioMedical Research, Cambridge, MA, United States | chris.odonnell@novartis.com | **Steering Committee** | **Pharmaceutical companies** |
| Nicole Renaud | Novartis Institutes for BioMedical Research, Cambridge, MA, United States | nicole.renaud@novartis.com | **Steering Committee** | **Pharmaceutical companies** |
| Tomi P. Mäkelä | HiLIFE, University of Helsinki, Finland, Finland | tomi.makela@helsinki.fi | **Steering Committee** | **University of Helsinki & Biobanks** |
| Jaakko Kaprio | Institute for Molecular Medicine Finland (FIMM), HiLIFE, University of Helsinki, Helsinki, Finland | jaakko.kaprio@helsinki.fi | **Steering Committee** | **University of Helsinki & Biobanks** |
| Petri Virolainen | Auria Biobank / University of Turku / Hospital District of Southwest Finland, Turku, Finland | petri.virolainen@tyks.fi | **Steering Committee** | **University of Helsinki & Biobanks** |
| Antti Hakanen | Auria Biobank / University of Turku / Hospital District of Southwest Finland, Turku, Finland | antti.hakanen@tyks.fi | **Steering Committee** | **University of Helsinki & Biobanks** |
| Terhi Kilpi | THL Biobank / Finnish Institute for Health and Welfare (THL), Helsinki, Finland | terhi.kilpi@thl.fi | **Steering Committee** | **University of Helsinki & Biobanks** |
| Markus Perola | THL Biobank / Finnish Institute for Health and Welfare (THL), Helsinki, Finland | markus.perola@thl.fi | **Steering Committee** | **University of Helsinki & Biobanks** |
| Jukka Partanen | Finnish Red Cross Blood Service / Finnish Hematology Registry and Clinical Biobank, Helsinki, Finland | jukka.partanen@veripalvelu.fi | **Steering Committee** | **University of Helsinki & Biobanks** |
| Anne Pitkäranta | Helsinki Biobank / Helsinki University and Hospital District of Helsinki and Uusimaa, Helsinki | anne.pitkaranta@hus.fi | **Steering Committee** | **University of Helsinki & Biobanks** |
| Taneli Raivio | Helsinki Biobank / Helsinki University and Hospital District of Helsinki and Uusimaa, Helsinki | taneli.raivio@hus.fi | **Steering Committee** | **University of Helsinki & Biobanks** |
| Jani Tikkanen | Northern Finland Biobank Borealis / University of Oulu / Northern Ostrobothnia Hospital District, Oulu, Finland | jani.tikkanen@ppshp.fi | **Steering Committee** | **University of Helsinki & Biobanks** |
| Raisa Serpi | Northern Finland Biobank Borealis / University of Oulu / Northern Ostrobothnia Hospital District, Oulu, Finland | raisa.serpi@ppshp.fi | **Steering Committee** | **University of Helsinki & Biobanks** |
| Tarja Laitinen | Finnish Clinical Biobank Tampere / University of Tampere / Pirkanmaa Hospital District, Tampere, Finland | tarja.laitinen@pshp.fi | **Steering Committee** | **University of Helsinki & Biobanks** |
| Veli-Matti Kosma | Biobank of Eastern Finland / University of Eastern Finland / Northern Savo Hospital District, Kuopio, Finland | veli-matti.kosma@uef.fi | **Steering Committee** | **University of Helsinki & Biobanks** |
| Jari Laukkanen | Central Finland Biobank / University of Jyväskylä / Central Finland Health Care District, Jyväskylä, Finland | jari.laukkanen@ksshp.fi | **Steering Committee** | **University of Helsinki & Biobanks** |
| Marco Hautalahti | FINBB - Finnish biobank cooperative | marco.hautalahti@finbb.fi | **Steering Committee** | **University of Helsinki & Biobanks** |
| Outi Tuovila | Business Finland, Helsinki, Finland | outi.tuovila@businessfinland.fi | **Steering Committee** | **Other Experts/ Non-Voting Members** |
| Raimo Pakkanen | Business Finland, Helsinki, Finland | raimo.pakkanen@businessfinland.fi | **Steering Committee** | **Other Experts/ Non-Voting Members** |
| Jeffrey Waring | Abbvie, Chicago, IL, United States | jeff.waring@abbvie.com | **Scientific Committee** | **Pharmaceutical companies** |
| Bridget Riley-Gillis | Abbvie, Chicago, IL, United States | bridget.rileygillis@abbvie.com | **Scientific Committee** | **Pharmaceutical companies** |
| Fedik Rahimov | Abbvie, Chicago, IL, United States | fedik.rahimov@abbvie.com | **Scientific Committee** | **Pharmaceutical companies** |
| Ioanna Tachmazidou | Astra Zeneca, Cambridge, United Kingdom | ioanna.tachmazidou@astrazeneca.com | **Scientific Committee** | **Pharmaceutical companies** |
| Chia-Yen Chen | Biogen, Cambridge, MA, United States | chiayen.chen@biogen.com | **Scientific Committee** | **Pharmaceutical companies** |
| Heiko Runz | Biogen, Cambridge, MA, United States | heiko.runz@biogen.com | **Scientific Committee** | **Pharmaceutical companies** |
| Zhihao Ding | Boehringer Ingelheim, Ingelheim am Rhein, Germany | zhihao.ding@boehringer-ingelheim.com | **Scientific Committee** | **Pharmaceutical companies** |
| Marc Jung | Boehringer Ingelheim, Ingelheim am Rhein, Germany | marc_oliver.jung@boehringer-ingelheim.com | **Scientific Committee** | **Pharmaceutical companies** |
| Hanati Tuoken | Boehringer Ingelheim, Ingelheim am Rhein, Germany | hanati.tuoken@boehringer-ingelheim.com | **Scientific Committee** | **Pharmaceutical companies** |
| Shameek Biswas | Bristol Myers Squibb, New York, NY, United States | Shameek.Biswas@bms.com | **Scientific Committee** | **Pharmaceutical companies** |
| Rion Pendergrass | Genentech, San Francisco, CA, United States | penders2@gene.com | **Scientific Committee** | **Pharmaceutical companies** |
| Margaret G. Ehm | GlaxoSmithKline, Collegeville, PA, United States | meg.g.ehm@gsk.com | **Scientific Committee** | **Pharmaceutical companies** |
| David Pulford | GlaxoSmithKline, Stevenage, United Kingdom | david.x.pulford@gsk.com | **Scientific Committee** | **Pharmaceutical companies** |
| Neha Raghavan | Merck, Kenilworth, NJ, United States | neha.raghavan@merck.com | **Scientific Committee** | **Pharmaceutical companies** |
| Adriana Huertas-Vazquez | Merck, Kenilworth, NJ, United States | adriana.huertas.vazquez@merck.com | **Scientific Committee** | **Pharmaceutical companies** |
| Jae-Hoon Sul | Merck, Kenilworth, NJ, United States | jae.hoon.sul@merck.com | **Scientific Committee** | **Pharmaceutical companies** |
| Anders Mälarstig | Pfizer, New York, NY, United States | anders.malarstig@pfizer.com | **Scientific Committee** | **Pharmaceutical companies** |
| Xinli Hu | Pfizer, New York, NY, United States | xinli.hu@pfizer.com | **Scientific Committee** | **Pharmaceutical companies** |
| Åsa Hedman | Pfizer, New York, NY, United States | asa.hedman@pfizer.com | **Scientific Committee** | **Pharmaceutical companies** |
| Katherine Klinger | Translational Sciences, Sanofi R&D, Framingham, MA, USA | katherine.klinger@sanofi.com | **Scientific Committee** | **Pharmaceutical companies** |
| Robert Graham | Maze Therapeutics, San Francisco, CA, United States | rgraham@mazetx.com | **Scientific Committee** | **Pharmaceutical companies** |
| Manuel Rivas | Maze Therapeutics, San Francisco, CA, United States | mrivas@mazetx.com | **Scientific Committee** | **Pharmaceutical companies** |
| Dawn Waterworth | Janssen Research & Development, LLC, Spring House, PA, United States | dwaterwo@its.jnj.com | **Scientific Committee** | **Pharmaceutical companies** |
| Nicole Renaud | Novartis Institutes for BioMedical Research, Cambridge, MA, United States | nicole.renaud@novartis.com | **Scientific Committee** | **Pharmaceutical companies** |
| Ma´en Obeidat | Novartis Institutes for BioMedical Research, Cambridge, MA, United States | maen.obeidat@novartis.com | **Scientific Committee** | **Pharmaceutical companies** |
| Jonathan Chung | Novartis Institutes for BioMedical Research, Cambridge, MA, United States | jonathan.chung@novartis.com | **Scientific Committee** | **Pharmaceutical companies** |
| Jonas Zierer | Novartis Institutes for BioMedical Research, Cambridge, MA, United States | jonas.zierer@novartis.com | **Scientific Committee** | **Pharmaceutical companies** |
| Mari Niemi | Novartis Institutes for BioMedical Research, Cambridge, MA, United States | mari.niemi@novartis.com | **Scientific Committee** | **Pharmaceutical companies** |
| Samuli Ripatti | Institute for Molecular Medicine Finland (FIMM), HiLIFE, University of Helsinki, Helsinki, Finland | samuli.ripatti@helsinki.fi | **Scientific Committee** | **University of Helsinki & Biobanks** |
| Johanna Schleutker | Auria Biobank / Univ. of Turku / Hospital District of Southwest Finland, Turku, Finland | johanna.schleutker@utu.fi | **Scientific Committee** | **University of Helsinki & Biobanks** |
| Markus Perola | THL Biobank / Finnish Institute for Health and Welfare (THL), Helsinki, Finland | markus.perola@thl.fi | **Scientific Committee** | **University of Helsinki & Biobanks** |
| Mikko Arvas | Finnish Red Cross Blood Service / Finnish Hematology Registry and Clinical Biobank, Helsinki, Finland | mikko.arvas@veripalvelu.fi | **Scientific Committee** | **University of Helsinki & Biobanks** |
| Olli Carpén | Helsinki Biobank / Helsinki University and Hospital District of Helsinki and Uusimaa, Helsinki | olli.carpen@helsinki.fi | **Scientific Committee** | **University of Helsinki & Biobanks** |
| Reetta Hinttala | Northern Finland Biobank Borealis / University of Oulu / Northern Ostrobothnia Hospital District, Oulu, Finland | reetta.hinttala@oulu.fi | **Scientific Committee** | **University of Helsinki & Biobanks** |
| Johannes Kettunen | Northern Finland Biobank Borealis / University of Oulu / Northern Ostrobothnia Hospital District, Oulu, Finland | johannes.kettunen@oulu.fi | **Scientific Committee** | **University of Helsinki & Biobanks** |
| Arto Mannermaa | Biobank of Eastern Finland / University of Eastern Finland / Northern Savo Hospital District, Kuopio, Finland | arto.mannermaa@uef.fi | **Scientific Committee** | **University of Helsinki & Biobanks** |
| Katriina Aalto-Setälä | Faculty of Medicine and Health Technology, Tampere University, Tampere, Finland | katriina.aalto-setala@tuni.fi | **Scientific Committee** | **University of Helsinki & Biobanks** |
| Mika Kähönen | Finnish Clinical Biobank Tampere / University of Tampere / Pirkanmaa Hospital District, Tampere, Finland | mika.kahonen@uta.fi | **Scientific Committee** | **University of Helsinki & Biobanks** |
| Jari Laukkanen | Central Finland Biobank / University of Jyväskylä / Central Finland Health Care District, Jyväskylä, Finland | jari.laukkanen@ksshp.fi | **Scientific Committee** | **University of Helsinki & Biobanks** |
| Johanna Mäkelä | FINBB - Finnish biobank cooperative | johanna.makela@finbb.fi | **Scientific Committee** | **University of Helsinki & Biobanks** |
| Reetta Kälviäinen | Northern Savo Hospital District, Kuopio, Finland | reetta.kalviainen@kuh.fi | **Clinical Groups** | **Neurology Group** |
| Valtteri Julkunen | Northern Savo Hospital District, Kuopio, Finland | valtteri.julkunen@kuh.fi | **Clinical Groups** | **Neurology Group** |
| Hilkka Soininen | Northern Savo Hospital District, Kuopio, Finland | hilkka.soininen@uef.fi | **Clinical Groups** | **Neurology Group** |
| Anne Remes | Northern Ostrobothnia Hospital District, Oulu, Finland | anne.remes@oulu.fi | **Clinical Groups** | **Neurology Group** |
| Mikko Hiltunen | University of Eastern Finland, Kuopio, Finland | mikko.hiltunen@uef.fi | **Clinical Groups** | **Neurology Group** |
| Jukka Peltola | Pirkanmaa Hospital District, Tampere, Finland | jukka.peltola@pshp.fi | **Clinical Groups** | **Neurology Group** |
| Minna Raivio | Hospital District of Helsinki and Uusimaa, Helsinki, Finland | minna.raivio@geri.fi | **Clinical Groups** | **Neurology Group** |
| Pentti Tienari | Hospital District of Helsinki and Uusimaa, Helsinki, Finland | pentti.tienari@hus.fi | **Clinical Groups** | **Neurology Group** |
| Juha Rinne | Hospital District of Southwest Finland, Turku, Finland | juha.rinne@tyks.fi | **Clinical Groups** | **Neurology Group** |
| Roosa Kallionpää | Hospital District of Southwest Finland, Turku, Finland | roosa.kallionpaa@tyks.fi | **Clinical Groups** | **Neurology Group** |
| Juulia Partanen | Institute for Molecular Medicine Finland, HiLIFE, University of Helsinki, Finland | juulia.partanen@helsinki.fi | **Clinical Groups** | **Neurology Group** |
| Adam Ziemann | Abbvie, Chicago, IL, United States | adam.ziemann@abbvie.com | **Clinical Groups** | **Neurology Group** |
| Nizar Smaoui | Abbvie, Chicago, IL, United States | nizar.smaoui@abbvie.com | **Clinical Groups** | **Neurology Group** |
| Anne Lehtonen | Abbvie, Chicago, IL, United States | anne.lehtonen@abbvie.com | **Clinical Groups** | **Neurology Group** |
| Susan Eaton | Biogen, Cambridge, MA, United States | susan.eaton@biogen.com | **Clinical Groups** | **Neurology Group** |
| Heiko Runz | Biogen, Cambridge, MA, United States | heiko.runz@biogen.com | **Clinical Groups** | **Neurology Group** |
| Sanni Lahdenperä | Biogen, Cambridge, MA, United States | sanni.lahdenpera@biogen.com | **Clinical Groups** | **Neurology Group** |
| Shameek Biswas | Bristol Myers Squibb, New York, NY, United States | shameek.biswas@bms.com | **Clinical Groups** | **Neurology Group** |
| Natalie Bowers | Genentech, San Francisco, CA, United States | bowersn1@gene.com | **Clinical Groups** | **Neurology Group** |
| Edmond Teng | Genentech, San Francisco, CA, United States | teng.edmond@gene.com | **Clinical Groups** | **Neurology Group** |
| Rion Pendergrass | Genentech, San Francisco, CA, United States | penders2@gene.com | **Clinical Groups** | **Neurology Group** |
| Fanli Xu | GlaxoSmithKline, Brentford, United Kingdom | chun-fang.2.xu@gsk.com | **Clinical Groups** | **Neurology Group** |
| David Pulford | GlaxoSmithKline, Stevenage, United Kingdom | david.x.pulford@gsk.com | **Clinical Groups** | **Neurology Group** |
| Kirsi Auro | GlaxoSmithKline, Espoo, Finland | kirsi.m.auro@gsk.com | **Clinical Groups** | **Neurology Group** |
| Laura Addis | GlaxoSmithKline, Brentford, United Kingdom | laura.x.addis@gsk.com | **Clinical Groups** | **Neurology Group** |
| John Eicher | GlaxoSmithKline, Brentford, United Kingdom | john.d.eicher@gsk.com | **Clinical Groups** | **Neurology Group** |
| Qingqin S Li | Janssen Research & Development, LLC, Titusville, NJ 08560, United States | QLi2@its.jnj.com | **Clinical Groups** | **Neurology Group** |
| Karen He | Janssen Research & Development, LLC, Spring House, PA, United States | khe2@its.jnj.com | **Clinical Groups** | **Neurology Group** |
| Ekaterina Khramtsova | Janssen Research & Development, LLC, Spring House, PA, United States | ekhramts@its.jnj.com | **Clinical Groups** | **Neurology Group** |
| Neha Raghavan | Merck, Kenilworth, NJ, United States | neha.raghavan@merck.com | **Clinical Groups** | **Neurology Group** |
| Martti Färkkilä | Hospital District of Helsinki and Uusimaa, Helsinki, Finland | martti.farkkila@hus.fi | **Clinical Groups** | **Gastroenterology Group** |
| Jukka Koskela | Hospital District of Helsinki and Uusimaa, Helsinki, Finland | jukka.koskela@helsinki.fi | **Clinical Groups** | **Gastroenterology Group** |
| Sampsa Pikkarainen | Hospital District of Helsinki and Uusimaa, Helsinki, Finland | sampsa.pikkarainen@hus.fi | **Clinical Groups** | **Gastroenterology Group** |
| Airi Jussila | Pirkanmaa Hospital District, Tampere, Finland | airi.jussila@pshp.fi | **Clinical Groups** | **Gastroenterology Group** |
| Katri Kaukinen | Pirkanmaa Hospital District, Tampere, Finland | katri.kaukinen@tuni.fi | **Clinical Groups** | **Gastroenterology Group** |
| Timo Blomster | Northern Ostrobothnia Hospital District, Oulu, Finland | timo.blomster@ppshp.fi | **Clinical Groups** | **Gastroenterology Group** |
| Mikko Kiviniemi | Northern Savo Hospital District, Kuopio, Finland | mikko.kiviniemi@kuh.fi | **Clinical Groups** | **Gastroenterology Group** |
| Markku Voutilainen | Hospital District of Southwest Finland, Turku, Finland | markku.voutilainen@tyks.fi | **Clinical Groups** | **Gastroenterology Group** |
| Mark Daly | Institute for Molecular Medicine, Finland (FIMM), HiLIFE, University of Helsinki, Helsinki, Finland; Broad Institute of MIT and Harvard; Massachusetts General Hospital | mark.daly@helsinki.fi | **Clinical Groups** | **Gastroenterology Group** |
| Jeffrey Waring | Abbvie, Chicago, IL, United States | jeff.waring@abbvie.com | **Clinical Groups** | **Gastroenterology Group** |
| Nizar Smaoui | Abbvie, Chicago, IL, United States | nizar.smaoui@abbvie.com | **Clinical Groups** | **Gastroenterology Group** |
| Fedik Rahimov | Abbvie, Chicago, IL, United States | fedik.rahimov@abbvie.com | **Clinical Groups** | **Gastroenterology Group** |
| Anne Lehtonen | Abbvie, Chicago, IL, United States | anne.lehtonen@abbvie.com | **Clinical Groups** | **Gastroenterology Group** |
| Tim Lu | Genentech, San Francisco, CA, United States | lut8@gene.com | **Clinical Groups** | **Gastroenterology Group** |
| Natalie Bowers | Genentech, San Francisco, CA, United States | bowersn1@gene.com | **Clinical Groups** | **Gastroenterology Group** |
| Rion Pendergrass | Genentech, San Francisco, CA, United States | penders2@gene.com | **Clinical Groups** | **Gastroenterology Group** |
| Linda McCarthy | GlaxoSmithKline, Brentford, United Kingdom | linda.c.mccarthy@gsk.com | **Clinical Groups** | **Gastroenterology Group** |
| Amy Hart | Janssen Research & Development, LLC, Spring House, PA, United States | ahart13@its.jnj.com | **Clinical Groups** | **Gastroenterology Group** |
| Meijian Guan | Janssen Research & Development, LLC, Spring House, PA, United States | mguan4@its.jnj.com | **Clinical Groups** | **Gastroenterology Group** |
| Jason Miller | Merck, Kenilworth, NJ, United States | jason.miller4@merck.com | **Clinical Groups** | **Gastroenterology Group** |
| Kirsi Kalpala | Pfizer, New York, NY, United States | kirsi.kalpala@pfizer.com | **Clinical Groups** | **Gastroenterology Group** |
| Melissa Miller | Pfizer, New York, NY, United States | melissa.r.miller@pfizer.com | **Clinical Groups** | **Gastroenterology Group** |
| Xinli Hu | Pfizer, New York, NY, United States | xinli.hu@pfizer.com | **Clinical Groups** | **Gastroenterology Group** |
| Kari Eklund | Hospital District of Helsinki and Uusimaa, Helsinki, Finland | kari.eklund@hus.fi | **Clinical Groups** | **Rheumatology Group** |
| Antti Palomäki | Hospital District of Southwest Finland, Turku, Finland | ajpalo@utu.fi | **Clinical Groups** | **Rheumatology Group** |
| Pia Isomäki | Pirkanmaa Hospital District, Tampere, Finland | pia.isomaki@pshp.fi | **Clinical Groups** | **Rheumatology Group** |
| Laura Pirilä | Hospital District of Southwest Finland, Turku, Finland | laura.pirila@fimnet.fi,laura.pirila@tyks.fi | **Clinical Groups** | **Rheumatology Group** |
| Oili Kaipiainen-Seppänen | Northern Savo Hospital District, Kuopio, Finland | oili.kaipiainen-seppanen@kuh.fi | **Clinical Groups** | **Rheumatology Group** |
| Johanna Huhtakangas | Northern Ostrobothnia Hospital District, Oulu, Finland | johanna.huhtakangas@kuh.fi | **Clinical Groups** | **Rheumatology Group** |
| Nina Mars | Institute for Molecular Medicine Finland (FIMM), HiLIFE, University of Helsinki, Helsinki, Finland | nina.mars@helsinki.fi | **Clinical Groups** | **Rheumatology Group** |
| Jeffrey Waring | Abbvie, Chicago, IL, United States | jeff.waring@abbvie.com | **Clinical Groups** | **Rheumatology Group** |
| Fedik Rahimov | Abbvie, Chicago, IL, United States | fedik.rahimov@abbvie.com | **Clinical Groups** | **Rheumatology Group** |
| Apinya Lertratanakul | Abbvie, Chicago, IL, United States | apinya.lertratanakul@abbvie.com | **Clinical Groups** | **Rheumatology Group** |
| Nizar Smaoui | Abbvie, Chicago, IL, United States | nizar.smaoui@abbvie.com | **Clinical Groups** | **Rheumatology Group** |
| Anne Lehtonen | Abbvie, Chicago, IL, United States | anne.lehtonen@abbvie.com | **Clinical Groups** | **Rheumatology Group** |
| Coralie Viollet | AstraZeneca, Cambridge, United Kingdom | coralie.viollet@astrazeneca.com | **Clinical Groups** | **Rheumatology Group** |
| Marla Hochfeld | Bristol Myers Squibb, New York, NY, United States | mhochfeld@celgene.com | **Clinical Groups** | **Rheumatology Group** |
| Natalie Bowers | Genentech, San Francisco, CA, United States | bowersn1@gene.com | **Clinical Groups** | **Rheumatology Group** |
| Rion Pendergrass | Genentech, San Francisco, CA, United States | penders2@gene.com | **Clinical Groups** | **Rheumatology Group** |
| Jorge Esparza Gordillo | GlaxoSmithKline, Brentford, United Kingdom | jorge.x.esparza-gordillo@gsk.com | **Clinical Groups** | **Rheumatology Group** |
| Kirsi Auro | GlaxoSmithKline, Espoo, Finland | kirsi.m.auro@gsk.com | **Clinical Groups** | **Rheumatology Group** |
| Dawn Waterworth | Janssen Research & Development, LLC, Spring House, PA, United States | dwaterwo@its.jnj.com | **Clinical Groups** | **Rheumatology Group** |
| Fabiana Farias | Merck, Kenilworth, NJ, United States | fabiana.farias@merck.com | **Clinical Groups** | **Rheumatology Group** |
| Kirsi Kalpala | Pfizer, New York, NY, United States | kirsi.kalpala@pfizer.com | **Clinical Groups** | **Rheumatology Group** |
| Nan Bing | Pfizer, New York, NY, United States | nan.bing@pfizer.com | **Clinical Groups** | **Rheumatology Group** |
| Xinli Hu | Pfizer, New York, NY, United States | xinli.hu@pfizer.com | **Clinical Groups** | **Rheumatology Group** |
| Tarja Laitinen | Pirkanmaa Hospital District, Tampere, Finland | tarja.laitinen@pshp.fi | **Clinical Groups** | **Pulmonology Group** |
| Margit Pelkonen | Northern Savo Hospital District, Kuopio, Finland | margit.pelkonen@kuh.fi | **Clinical Groups** | **Pulmonology Group** |
| Paula Kauppi | Hospital District of Helsinki and Uusimaa, Helsinki, Finland | paula.kauppi@hus.fi | **Clinical Groups** | **Pulmonology Group** |
| Hannu Kankaanranta | University of Gothenburg, Gothenburg, Sweden/ Seinäjoki Central Hospital, Seinäjoki, Finland/ Tampere University, Tampere, Finland | hannu.kankaanranta@tuni.fi | **Clinical Groups** | **Pulmonology Group** |
| Terttu Harju | Northern Ostrobothnia Hospital District, Oulu, Finland | terttu.harju@oulu.fi | **Clinical Groups** | **Pulmonology Group** |
| Riitta Lahesmaa | Hospital District of Southwest Finland, Turku, Finland | rilahes@utu.fi | **Clinical Groups** | **Pulmonology Group** |
| Nizar Smaoui | Abbvie, Chicago, IL, United States | nizar.smaoui@abbvie.com | **Clinical Groups** | **Pulmonology Group** |
| Coralie Viollet | AstraZeneca, Cambridge, United Kingdom | coralie.viollet@astrazeneca.com | **Clinical Groups** | **Pulmonology Group** |
| Susan Eaton | Biogen, Cambridge, MA, United States | susan.eaton@biogen.com | **Clinical Groups** | **Pulmonology Group** |
| Hubert Chen | Genentech, San Francisco, CA, United States | chenh37@gene.com | **Clinical Groups** | **Pulmonology Group** |
| Rion Pendergrass | Genentech, San Francisco, CA, United States | penders2@gene.com | **Clinical Groups** | **Pulmonology Group** |
| Natalie Bowers | Genentech, San Francisco, CA, United States | bowersn1@gene.com | **Clinical Groups** | **Pulmonology Group** |
| Joanna Betts | GlaxoSmithKline, Brentford, United Kingdom | joanna.c.betts@gsk.com | **Clinical Groups** | **Pulmonology Group** |
| Kirsi Auro | GlaxoSmithKline, Espoo, Finland | kirsi.m.auro@gsk.com | **Clinical Groups** | **Pulmonology Group** |
| Rajashree Mishra | GlaxoSmithKline, Brentford, United Kingdom | rajashree.x.mishra@gsk.com | **Clinical Groups** | **Pulmonology Group** |
| Majd Mouded | Novartis, Basel, Switzerland | majd.mouded@novartis.com | **Clinical Groups** | **Pulmonology Group** |
| Debby Ngo | Novartis, Basel, Switzerland | debby.ngo@novartis.com | **Clinical Groups** | **Pulmonology Group** |
| Teemu Niiranen | Finnish Institute for Health and Welfare (THL), Helsinki, Finland | teemu.niiranen@thl.fi | **Clinical Groups** | **Cardiometabolic Diseases Group** |
| Felix Vaura | Finnish Institute for Health and Welfare (THL), Helsinki, Finland | fechva@utu.fi | **Clinical Groups** | **Cardiometabolic Diseases Group** |
| Veikko Salomaa | Finnish Institute for Health and Welfare (THL), Helsinki, Finland | veikko.salomaa@thl.fi | **Clinical Groups** | **Cardiometabolic Diseases Group** |
| Kaj Metsärinne | Hospital District of Southwest Finland, Turku, Finland | kaj.metsarinne@tyks.fi | **Clinical Groups** | **Cardiometabolic Diseases Group** |
| Jenni Aittokallio | Hospital District of Southwest Finland, Turku, Finland | jemato@utu.fi | **Clinical Groups** | **Cardiometabolic Diseases Group** |
| Mika Kähönen | Pirkanmaa Hospital District, Tampere, Finland | mika.kahonen@uta.fi | **Clinical Groups** | **Cardiometabolic Diseases Group** |
| Jussi Hernesniemi | Pirkanmaa Hospital District, Tampere, Finland | jussi.hernesniemi@tuni.fi | **Clinical Groups** | **Cardiometabolic Diseases Group** |
| Daniel Gordin | Hospital District of Helsinki and Uusimaa, Helsinki, Finland | daniel.gordin@hus.fi | **Clinical Groups** | **Cardiometabolic Diseases Group** |
| Juha Sinisalo | Hospital District of Helsinki and Uusimaa, Helsinki, Finland | juha.sinisalo@hus.fi | **Clinical Groups** | **Cardiometabolic Diseases Group** |
| Marja-Riitta Taskinen | Hospital District of Helsinki and Uusimaa, Helsinki, Finland | marja-riitta.taskinen@helsinki.fi | **Clinical Groups** | **Cardiometabolic Diseases Group** |
| Tiinamaija Tuomi | Hospital District of Helsinki and Uusimaa, Helsinki, Finland | tiinamaija.tuomi@hus.fi | **Clinical Groups** | **Cardiometabolic Diseases Group** |
| Timo Hiltunen | Hospital District of Helsinki and Uusimaa, Helsinki, Finland | timo.hiltunen@hus.fi | **Clinical Groups** | **Cardiometabolic Diseases Group** |
| Jari Laukkanen | Central Finland Health Care District, Jyväskylä, Finland | jari.laukkanen@ksshp.fi | **Clinical Groups** | **Cardiometabolic Diseases Group** |
| Amanda Elliott | Institute for Molecular Medicine Finland (FIMM), HiLIFE, University of Helsinki, Helsinki, Finland; Broad Institute, Cambridge, MA, USA and Massachusetts General Hospital, Boston, MA, USA | aelliott@broadinstitute.org | **Clinical Groups** | **Cardiometabolic Diseases Group** |
| Mary Pat Reeve | Institute for Molecular Medicine Finland (FIMM), HiLIFE, University of Helsinki, Helsinki, Finland | mary.reeve@helsinki.fi | **Clinical Groups** | **Cardiometabolic Diseases Group** |
| Sanni Ruotsalainen | Institute for Molecular Medicine Finland (FIMM), HiLIFE, University of Helsinki, Helsinki, Finland | sanni.ruotsalainen@helsinki.fi | **Clinical Groups** | **Cardiometabolic Diseases Group** |
| Dirk Paul | Astra Zeneca, Cambridge, United Kingdom | dirk.paul@astrazeneca.com | **Clinical Groups** | **Cardiometabolic Diseases Group** |
| Natalie Bowers | Genentech, San Francisco, CA, United States | bowersn1@gene.com | **Clinical Groups** | **Cardiometabolic Diseases Group** |
| Rion Pendergrass | Genentech, San Francisco, CA, United States | penders2@gene.com | **Clinical Groups** | **Cardiometabolic Diseases Group** |
| Audrey Chu | GlaxoSmithKline, Brentford, United Kingdom | audrey.y.chu@gsk.com | **Clinical Groups** | **Cardiometabolic Diseases Group** |
| Kirsi Auro | GlaxoSmithKline, Espoo, Finland | kirsi.m.auro@gsk.com | **Clinical Groups** | **Cardiometabolic Diseases Group** |
| Dermot Reilly | Janssen Research & Development, LLC, Boston, MA, United States | dreill11@its.jnj.com | **Clinical Groups** | **Cardiometabolic Diseases Group** |
| Mike Mendelson | Novartis, Boston, MA, United States | mike.mendelson@novartis.com | **Clinical Groups** | **Cardiometabolic Diseases Group** |
| Jaakko Parkkinen | Pfizer, New York, NY, United States | jaakko.parkkinen@pfizer.com | **Clinical Groups** | **Cardiometabolic Diseases Group** |
| Melissa Miller | Pfizer, New York, NY, United States | melissa.r.miller@pfizer.com | **Clinical Groups** | **Cardiometabolic Diseases Group** |
| Tuomo Meretoja | Hospital District of Helsinki and Uusimaa, Helsinki, Finland | tuomo.meretoja@hus.fi | **Clinical Groups** | **Oncology Group** |
| Heikki Joensuu | Hospital District of Helsinki and Uusimaa, Helsinki, Finland | heikki.joensuu@hus.fi | **Clinical Groups** | **Oncology Group** |
| Olli Carpén | Hospital District of Helsinki and Uusimaa, Helsinki, Finland | olli.carpen@helsinki.fi | **Clinical Groups** | **Oncology Group** |
| Johanna Mattson | Hospital District of Helsinki and Uusimaa, Helsinki, Finland | johanna.mattson@hus.fi | **Clinical Groups** | **Oncology Group** |
| Eveliina Salminen | Hospital District of Helsinki and Uusimaa, Helsinki, Finland | eveliina.e.salminen@hus.fi | **Clinical Groups** | **Oncology Group** |
| Annika Auranen | Pirkanmaa Hospital District , Tampere, Finland | anaura@utu.fi | **Clinical Groups** | **Oncology Group** |
| Peeter Karihtala | Northern Ostrobothnia Hospital District, Oulu, Finland | peeter.karihtala@oulu.fi | **Clinical Groups** | **Oncology Group** |
| Päivi Auvinen | Northern Savo Hospital District, Kuopio, Finland | paivi.auvinen@kuh.fi | **Clinical Groups** | **Oncology Group** |
| Klaus Elenius | Hospital District of Southwest Finland, Turku, Finland | klaus.elenius@utu.fi | **Clinical Groups** | **Oncology Group** |
| Johanna Schleutker | Hospital District of Southwest Finland, Turku, Finland | johanna.schleutker@utu.fi | **Clinical Groups** | **Oncology Group** |
| Esa Pitkänen | Institute for Molecular Medicine Finland (FIMM), HiLIFE, University of Helsinki, Helsinki, Finland | esa.pitkanen@helsinki.fi | **Clinical Groups** | **Oncology Group** |
| Nina Mars | Institute for Molecular Medicine Finland (FIMM), HiLIFE, University of Helsinki, Helsinki, Finland | nina.mars@helsinki.fi | **Clinical Groups** | **Oncology Group** |
| Mark Daly | Institute for Molecular Medicine Finland (FIMM), HiLIFE, University of Helsinki, Helsinki, Finland; Broad Institute of MIT and Harvard; Massachusetts General Hospital | mark.daly@helsinki.fi | **Clinical Groups** | **Oncology Group** |
| Relja Popovic | Abbvie, Chicago, IL, United States | relja.popovic@abbvie.com | **Clinical Groups** | **Oncology Group** |
| Jeffrey Waring | Abbvie, Chicago, IL, United States | jeff.waring@abbvie.com | **Clinical Groups** | **Oncology Group** |
| Bridget Riley-Gillis | Abbvie, Chicago, IL, United States | bridget.rileygillis@abbvie.com | **Clinical Groups** | **Oncology Group** |
| Anne Lehtonen | Abbvie, Chicago, IL, United States | anne.lehtonen@abbvie.com | **Clinical Groups** | **Oncology Group** |
| Margarete Fabre | AstraZeneca, Cambridge, United Kingdom | margarete.fabre@astrazeneca.com | **Clinical Groups** | **Oncology Group** |
| Jennifer Schutzman | Genentech, San Francisco, CA, United States | schutzman.jennifer@gene.com | **Clinical Groups** | **Oncology Group** |
| Natalie Bowers | Genentech, San Francisco, CA, United States | bowersn1@gene.com | **Clinical Groups** | **Oncology Group** |
| Rion Pendergrass | Genentech, San Francisco, CA, United States | penders2@gene.com | **Clinical Groups** | **Oncology Group** |
| Diptee Kulkarni | GlaxoSmithKline, Brentford, United Kingdom | diptee.a.kulkarni@gsk.com | **Clinical Groups** | **Oncology Group** |
| Kirsi Auro | GlaxoSmithKline, Espoo, Finland | kirsi.m.auro@gsk.com | **Clinical Groups** | **Oncology Group** |
| Alessandro Porello | Janssen Research & Development, LLC, Spring House, PA, United States | APorrell@ITS.JNJ.com | **Clinical Groups** | **Oncology Group** |
| Andrey Loboda | Merck, Kenilworth, NJ, United States | andrey_loboda@merck.com | **Clinical Groups** | **Oncology Group** |
| Heli Lehtonen | Pfizer, New York, NY, United States | heli.lehtonen@pfizer.com | **Clinical Groups** | **Oncology Group** |
| Stefan McDonough | Pfizer, New York, NY, United States | stefan.McDonough@pfizer.com | **Clinical Groups** | **Oncology Group** |
| Sauli Vuoti | Janssen-Cilag Oy, Espoo, Finland | svuoti@its.jnj.com | **Clinical Groups** | **Oncology Group** |
| Kai Kaarniranta | Northern Savo Hospital District, Kuopio, Finland; Department of Molecular Genetics, University of Lodz, Lodz, Poland | kai.kaarniranta@uef.fi | **Clinical Groups** | **Opthalmology Group** |
| Joni A Turunen | Helsinki University Hospital and University of Helsinki, Helsinki, Finland; Eye Genetics Group, Folkhälsan Research Center, Helsinki, Finland | joni.turunen@helsinki.fi | **Clinical Groups** | **Opthalmology Group** |
| Terhi Ollila | Hospital District of Helsinki and Uusimaa, Helsinki, Finland | terhi.ollila@hus.fi | **Clinical Groups** | **Opthalmology Group** |
| Hannu Uusitalo | Pirkanmaa Hospital District, Tampere, Finland | hannu.uusitalo@tuni.fi | **Clinical Groups** | **Opthalmology Group** |
| Juha Karjalainen | Institute for Molecular Medicine Finland (FIMM), HiLIFE, University of Helsinki, Helsinki, Finland | juha.karjalainen@helsinki.fi | **Clinical Groups** | **Opthalmology Group** |
| Esa Pitkänen | Institute for Molecular Medicine Finland (FIMM), HiLIFE, University of Helsinki, Helsinki, Finland | esa.pitkanen@helsinki.fi | **Clinical Groups** | **Opthalmology Group** |
| Mengzhen Liu | Abbvie, Chicago, IL, United States | mengzhen.liu@abbvie.com | **Clinical Groups** | **Opthalmology Group** |
| Heiko Runz | Biogen, Cambridge, MA, United States | heiko.runz@biogen.com | **Clinical Groups** | **Opthalmology Group** |
| Stephanie Loomis | Biogen, Cambridge, MA, United States | stephanie.loomis@biogen.com | **Clinical Groups** | **Opthalmology Group** |
| Erich Strauss | Genentech, San Francisco, CA, United States | strauss.erich@gene.com | **Clinical Groups** | **Opthalmology Group** |
| Natalie Bowers | Genentech, San Francisco, CA, United States | bowersn1@gene.com | **Clinical Groups** | **Opthalmology Group** |
| Hao Chen | Genentech, San Francisco, CA, United States | haoc@gene.com | **Clinical Groups** | **Opthalmology Group** |
| Rion Pendergrass | Genentech, San Francisco, CA, United States | penders2@gene.com | **Clinical Groups** | **Opthalmology Group** |
| Kaisa Tasanen | Northern Ostrobothnia Hospital District, Oulu, Finland | kaisa.tasanen-maatta@oulu.fi | **Clinical Groups** | **Dermatology Group** |
| Laura Huilaja | Northern Ostrobothnia Hospital District, Oulu, Finland | laura.huilaja@oulu.fi | **Clinical Groups** | **Dermatology Group** |
| Katariina Hannula-Jouppi | Hospital District of Helsinki and Uusimaa, Helsinki, Finland | katariina.hannula-jouppi@hus.fi | **Clinical Groups** | **Dermatology Group** |
| Teea Salmi | Pirkanmaa Hospital District, Tampere, Finland | teea.salmi@pshp.fi | **Clinical Groups** | **Dermatology Group** |
| Sirkku Peltonen | Hospital District of Southwest Finland, Turku, Finland | sipelto@utu.fi | **Clinical Groups** | **Dermatology Group** |
| Leena Koulu | Hospital District of Southwest Finland, Turku, Finland | leena.koulu@tyks.fi | **Clinical Groups** | **Dermatology Group** |
| Nizar Smaoui | Abbvie, Chicago, IL, United States | nizar.smaoui@abbvie.com | **Clinical Groups** | **Dermatology Group** |
| Fedik Rahimov | Abbvie, Chicago, IL, United States | fedik.rahimov@abbvie.com | **Clinical Groups** | **Dermatology Group** |
| Anne Lehtonen | Abbvie, Chicago, IL, United States | anne.lehtonen@abbvie.com | **Clinical Groups** | **Dermatology Group** |
| David Choy | Genentech, San Francisco, CA, United States | choy.david@gene.com | **Clinical Groups** | **Dermatology Group** |
| Rion Pendergrass | Genentech, San Francisco, CA, United States | penders2@gene.com | **Clinical Groups** | **Dermatology Group** |
| Dawn Waterworth | Janssen Research & Development, LLC, Spring House, PA, United States | dwaterwo@its.jnj.com | **Clinical Groups** | **Dermatology Group** |
| Kirsi Kalpala | Pfizer, New York, NY, United States | kirsi.kalpala@pfizer.com | **Clinical Groups** | **Dermatology Group** |
| Ying Wu | Pfizer, New York, NY, United States | ying.wu3@pfizer.com | **Clinical Groups** | **Dermatology Group** |
| Pirkko Pussinen | Hospital District of Helsinki and Uusimaa, Helsinki, Finland | pirkko.pussinen@helsinki.fi | **Clinical Groups** | **Odontology Group** |
| Aino Salminen | Hospital District of Helsinki and Uusimaa, Helsinki, Finland | aino.m.salminen@helsinki.fi | **Clinical Groups** | **Odontology Group** |
| Tuula Salo | Hospital District of Helsinki and Uusimaa, Helsinki, Finland | tuula.salo@helsinki.fi | **Clinical Groups** | **Odontology Group** |
| David Rice | Hospital District of Helsinki and Uusimaa, Helsinki, Finland | david.rice@helsinki.fi | **Clinical Groups** | **Odontology Group** |
| Pekka Nieminen | Hospital District of Helsinki and Uusimaa, Helsinki, Finland | pekka.nieminen@helsinki.fi | **Clinical Groups** | **Odontology Group** |
| Ulla Palotie | Hospital District of Helsinki and Uusimaa, Helsinki, Finland | ulla.palotie@helsinki.fi | **Clinical Groups** | **Odontology Group** |
| Maria Siponen | Northern Savo Hospital District, Kuopio, Finland | maria.siponen@uef.fi | **Clinical Groups** | **Odontology Group** |
| Liisa Suominen | Northern Savo Hospital District, Kuopio, Finland | liisa.suominen@uef.fi | **Clinical Groups** | **Odontology Group** |
| Päivi Mäntylä | Northern Savo Hospital District, Kuopio, Finland | paivi.mantyla@uef.fi | **Clinical Groups** | **Odontology Group** |
| Ulvi Gursoy | Hospital District of Southwest Finland, Turku, Finland | ulvi.gursoy@utu.fi | **Clinical Groups** | **Odontology Group** |
| Vuokko Anttonen | Northern Ostrobothnia Hospital District, Oulu, Finland | vuokko.anttonen@oulu.fi | **Clinical Groups** | **Odontology Group** |
| Kirsi Sipilä | Research Unit of Oral Health Sciences Faculty of Medicine, University of Oulu, Oulu, Finland; Medical Research Center, Oulu, Oulu University Hospital and University of Oulu, Oulu, Finland | kirsi.sipila@oulu.fi | **Clinical Groups** | **Odontology Group** |
| Rion Pendergrass | Genentech, San Francisco, CA, United States | pendergrass.sarah@gene.com | **Clinical Groups** | **Odontology Group** |
| Hannele Laivuori | Institute for Molecular Medicine Finland (FIMM), HiLIFE, University of Helsinki, Helsinki, Finland | hannele.laivuori@helsinki.fi | **Clinical Groups** | **Women’s Health and Reproduction Group** |
| Venla Kurra | Pirkanmaa Hospital District, Tampere, Finland | venla.kurra@tuni.fi | **Clinical Groups** | **Women’s Health and Reproduction Group** |
| Laura Kotaniemi-Talonen | Pirkanmaa Hospital District, Tampere, Finland | laura.kotaniemi-talonen@tuni.fi | **Clinical Groups** | **Women’s Health and Reproduction Group** |
| Oskari Heikinheimo | Hospital District of Helsinki and Uusimaa, Helsinki, Finland | oskari.heikinheimo@helsinki.fi | **Clinical Groups** | **Women’s Health and Reproduction Group** |
| Ilkka Kalliala | Hospital District of Helsinki and Uusimaa, Helsinki, Finland | ilkka.kalliala@hus.fi | **Clinical Groups** | **Women’s Health and Reproduction Group** |
| Lauri Aaltonen | Hospital District of Helsinki and Uusimaa, Helsinki, Finland | lauri.aaltonen@helsinki.fi | **Clinical Groups** | **Women’s Health and Reproduction Group** |
| Varpu Jokimaa | Hospital District of Southwest Finland, Turku, Finland | varpu.jokimaa@utu.fi | **Clinical Groups** | **Women’s Health and Reproduction Group** |
| Johannes Kettunen | Northern Ostrobothnia Hospital District, Oulu, Finland | Johannes.Kettunen@oulu.fi | **Clinical Groups** | **Women’s Health and Reproduction Group** |
| Marja Vääräsmäki | Northern Ostrobothnia Hospital District, Oulu, Finland | marja.vaarasmaki@oulu.fi | **Clinical Groups** | **Women’s Health and Reproduction Group** |
| Outi Uimari | Northern Ostrobothnia Hospital District, Oulu, Finland | outi.uimari@oulu.fi | **Clinical Groups** | **Women’s Health and Reproduction Group** |
| Laure Morin-Papunen | Northern Ostrobothnia Hospital District, Oulu, Finland | lmp@cc.oulu.fi | **Clinical Groups** | **Women’s Health and Reproduction Group** |
| Maarit Niinimäki | Northern Ostrobothnia Hospital District, Oulu, Finland | maarit.niinimaki@oulu.fi | **Clinical Groups** | **Women’s Health and Reproduction Group** |
| Terhi Piltonen | Northern Ostrobothnia Hospital District, Oulu, Finland | terhi.piltonen@oulu.fi | **Clinical Groups** | **Women’s Health and Reproduction Group** |
| Katja Kivinen | Institute for Molecular Medicine Finland (FIMM), HiLIFE, University of Helsinki, Helsinki, Finland | katja.kivinen@helsinki.fi | **Clinical Groups** | **Women’s Health and Reproduction Group** |
| Elisabeth Widen | Institute for Molecular Medicine Finland (FIMM), HiLIFE, University of Helsinki, Helsinki, Finland | elisabeth.widen@helsinki.fi | **Clinical Groups** | **Women’s Health and Reproduction Group** |
| Taru Tukiainen | Institute for Molecular Medicine Finland (FIMM), HiLIFE, University of Helsinki, Helsinki, Finland | taru.tukiainen@helsinki.fi | **Clinical Groups** | **Women’s Health and Reproduction Group** |
| Mary Pat Reeve | Institute for Molecular Medicine Finland (FIMM), HiLIFE, University of Helsinki, Helsinki, Finland | mary.reeve@helsinki.fi | **Clinical Groups** | **Women’s Health and Reproduction Group** |
| Mark Daly | Institute for Molecular Medicine Finland (FIMM), HiLIFE, University of Helsinki, Helsinki, Finland; Broad Institute of MIT and Harvard; Massachusetts General Hospital | mark.daly@helsinki.fi | **Clinical Groups** | **Women’s Health and Reproduction Group** |
| Niko Välimäki | University of Helsinki, Helsinki, Finland | niko.valimaki@helsinki.fi | **Clinical Groups** | **Women’s Health and Reproduction Group** |
| Eija Laakkonen | University of Jyväskylä, Jyväskylä, Finland | eija.k.laakkonen@jyu.fi | **Clinical Groups** | **Women’s Health and Reproduction Group** |
| Jaakko Tyrmi | University of Oulu, Oulu, Finland / University of Tampere, Tampere, Finland | jaakko.tyrmi@oulu.fi | **Clinical Groups** | **Women’s Health and Reproduction Group** |
| Heidi Silven | University of Oulu, Oulu, Finland | heidi.silven@student.oulu.fi | **Clinical Groups** | **Women’s Health and Reproduction Group** |
| Eeva Sliz | University of Oulu, Oulu, Finland | eeva.sliz@oulu.fi | **Clinical Groups** | **Women’s Health and Reproduction Group** |
| Riikka Arffman | University of Oulu, Oulu, Finland | riikka.arffman@oulu.fi | **Clinical Groups** | **Women’s Health and Reproduction Group** |
| Susanna Savukoski | University of Oulu, Oulu, Finland | susanna.savukoski@oulu.fi | **Clinical Groups** | **Women’s Health and Reproduction Group** |
| Triin Laisk | Estonian biobank, Tartu, Estonia | triin.laisk@ut.ee | **Clinical Groups** | **Women’s Health and Reproduction Group** |
| Natalia Pujol | Estonian biobank, Tartu, Estonia | natalia.pujolgualdo@oulu.fi | **Clinical Groups** | **Women’s Health and Reproduction Group** |
| Mengzhen Liu | Abbvie, Chicago, IL, United States | mengzhen.liu@abbvie.com | **Clinical Groups** | **Women’s Health and Reproduction Group** |
| Bridget Riley-Gillis | Abbvie, Chicago, IL, United States | bridget.rileygillis@abbvie.com | **Clinical Groups** | **Women’s Health and Reproduction Group** |
| Rion Pendergrass | Genentech, San Francisco, CA, United States | penders2@gene.com | **Clinical Groups** | **Women’s Health and Reproduction Group** |
| Janet Kumar | GlaxoSmithKline, Collegeville, PA, United States | janet.x.kumar@gsk.com | **Clinical Groups** | **Women’s Health and Reproduction Group** |
| Kirsi Auro | GlaxoSmithKline, Espoo, Finland | kirsi.m.auro@gsk.com | **Clinical Groups** | **Women’s Health and Reproduction Group** |
| Iiris Hovatta | University of Helsinki, Finland | iiris.hovatta@helsinki.fi | **Clinical Groups** | **Depression group** |
| Chia-Yen Chen | Biogen, Cambridge, MA, United States | chiayen.chen@biogen.com | **Clinical Groups** | **Depression group** |
| Erkki Isometsä | Hospital District of Helsinki and Uusimaa, Helsinki, Finland | erkki.isometsa@hus.fi | **Clinical Groups** | **Depression group** |
| Hanna Ollila | Institute for Molecular Medicine Finland (FIMM), HiLIFE, University of Helsinki, Helsinki, Finland | hanna.m.ollila@helsinki.fi | **Clinical Groups** | **Depression group** |
| Jaana Suvisaari | Finnish Institute for Health and Welfare (THL), Helsinki, Finland | jaana.suvisaari@thl.fi | **Clinical Groups** | **Depression group** |
| Antti Mäkitie | Department of Otorhinolaryngology - Head and Neck Surgery, University of Helsinki and Helsinki University Hospital, Helsinki, Finland | antti.makitie@helsinki.fi | **Clinical Groups** | **ENT (ear, nose and throath) Group** |
| Argyro Bizaki-Vallaskangas | Pirkanmaa Hospital District, Tampere, Finland | argyro.bizaki-vallaskangas@tuni.fi | **Clinical Groups** | **ENT (ear, nose and throath) Group** |
| Sanna Toppila-Salmi | University of Eastern Finland and Kuopio University Hospital, Department of Otorhinolaryngology, Kuopio, Finland and Department of Allergy, Helsinki University Hospital and University of Helsinki, Finland | sanna.salmi@helsinki.fi | **Clinical Groups** | **ENT (ear, nose and throath) Group** |
| Tytti Willberg | Hospital District of Southwest Finland, Turku, Finland | tytti.willberg@tyks.fi | **Clinical Groups** | **ENT (ear, nose and throath) Group** |
| Elmo Saarentaus | Institute for Molecular Medicine Finland (FIMM), HiLIFE, University of Helsinki, Helsinki, Finland | elmo.saarentaus@helsinki.fi | **Clinical Groups** | **ENT (ear, nose and throath) Group** |
| Antti Aarnisalo | Hospital District of Helsinki and Uusimaa, Helsinki, Finland | antti.aarnisalo@hus.fi | **Clinical Groups** | **ENT (ear, nose and throath) Group** |
| Eveliina Salminen | Hospital District of Helsinki and Uusimaa, Helsinki, Finland | eveliina.e.salminen@hus.fi | **Clinical Groups** | **ENT (ear, nose and throath) Group** |
| Elisa Rahikkala | Northern Ostrobothnia Hospital District, Oulu, Finland | elisa.rahikkala@ppshp.fi | **Clinical Groups** | **ENT (ear, nose and throath) Group** |
| Johannes Kettunen | Northern Ostrobothnia Hospital District, Oulu, Finland | johannes.kettunen@oulu.fi | **Clinical Groups** | **ENT (ear, nose and throath) Group** |
| Kristiina Aittomäki | Department of Medical Genetics, Helsinki University Central Hospital, Helsinki, Finland | kristiina.aittomaki@helsinki.fi | **Clinical Groups** | **POI (premature ovarian failure) Group** |
| Fredrik Åberg | Transplantation and Liver Surgery Clinic, Helsinki University Hospital, Helsinki University, Helsinki, Finland | fredrik.aberg@helsinki.fi | **Clinical Groups** | **LiverScore Group** |
| Mitja Kurki | Institute for Molecular Medicine Finland (FIMM), HiLIFE, University of Helsinki, Helsinki, Finland; Broad Institute, Cambridge, MA, United States | mkurki@broadinstitute.org | **FinnGen Analysis working group** | **FinnGen Analysis working group** |
| Samuli Ripatti | Institute for Molecular Medicine Finland (FIMM), HiLIFE, University of Helsinki, Helsinki, Finland | samuli.ripatti@helsinki.fi | **FinnGen Analysis working group** | **FinnGen Analysis working group** |
| Mark Daly | Institute for Molecular Medicine, Finland (FIMM), HiLIFE, University of Helsinki, Helsinki, Finland; Broad Institute of MIT and Harvard; Massachusetts General Hospital | mark.daly@helsinki.fi | **FinnGen Analysis working group** | **FinnGen Analysis working group** |
| Juha Karjalainen | Institute for Molecular Medicine Finland (FIMM), HiLIFE, University of Helsinki, Helsinki, Finland | juha.karjalainen@helsinki.fi | **FinnGen Analysis working group** | **FinnGen Analysis working group** |
| Aki Havulinna | Institute for Molecular Medicine Finland (FIMM), HiLIFE, University of Helsinki, Helsinki, Finland; Finnish Institute for Health and Welfare (THL), Helsinki, Finland | aki.havulinna@helsinki.fi | **FinnGen Analysis working group** | **FinnGen Analysis working group** |
| Juha Mehtonen | Institute for Molecular Medicine Finland (FIMM), HiLIFE, University of Helsinki, Helsinki, Finland | juha.mehtonen@helsinki.fi | **FinnGen Analysis working group** | **FinnGen Analysis working group** |
| Priit Palta | Institute for Molecular Medicine Finland (FIMM), HiLIFE, University of Helsinki, Helsinki, Finland | priit.palta@helsinki.fi | **FinnGen Analysis working group** | **FinnGen Analysis working group** |
| Shabbeer Hassan | Institute for Molecular Medicine Finland (FIMM), HiLIFE, University of Helsinki, Helsinki, Finland | shabbeer.hassan@helsinki.fi | **FinnGen Analysis working group** | **FinnGen Analysis working group** |
| Pietro Della Briotta Parolo | Institute for Molecular Medicine Finland (FIMM), HiLIFE, University of Helsinki, Helsinki, Finland | pietro.dellabriottaparolo@helsinki.fi | **FinnGen Analysis working group** | **FinnGen Analysis working group** |
| Wei Zhou | Broad Institute, Cambridge, MA, United States | wzhou@broadinstitute.org | **FinnGen Analysis working group** | **FinnGen Analysis working group** |
| Mutaamba Maasha | Broad Institute, Cambridge, MA, United States | mmaasha@broadinstitute.org | **FinnGen Analysis working group** | **FinnGen Analysis working group** |
| Shabbeer Hassan | Institute for Molecular Medicine Finland (FIMM), HiLIFE, University of Helsinki, Helsinki, Finland | shabbeer.hassan@helsinki.fi | **FinnGen Analysis working group** | **FinnGen Analysis working group** |
| Susanna Lemmelä | Institute for Molecular Medicine Finland (FIMM), HiLIFE, University of Helsinki, Helsinki, Finland | susanna.lemmela@helsinki.fi | **FinnGen Analysis working group** | **FinnGen Analysis working group** |
| Manuel Rivas | University of Stanford, Stanford, CA, United States | mrivas@stanford.edu | **FinnGen Analysis working group** | **FinnGen Analysis working group** |
| Aarno Palotie | Institute for Molecular Medicine Finland (FIMM), HiLIFE, University of Helsinki, Helsinki, Finland | aarno.palotie@helsinki.fi | **FinnGen Analysis working group** | **FinnGen Analysis working group** |
| Aoxing Liu | Institute for Molecular Medicine Finland (FIMM), HiLIFE, University of Helsinki, Helsinki, Finland | aoxing.liu@helsinki.fi | **FinnGen Analysis working group** | **FinnGen Analysis working group** |
| Arto Lehisto | Institute for Molecular Medicine Finland (FIMM), HiLIFE, University of Helsinki, Helsinki, Finland | arto.lehisto@helsinki.fi | **FinnGen Analysis working group** | **FinnGen Analysis working group** |
| Andrea Ganna | Institute for Molecular Medicine Finland (FIMM), HiLIFE, University of Helsinki, Helsinki, Finland | aganna@broadinstitute.org | **FinnGen Analysis working group** | **FinnGen Analysis working group** |
| Vincent Llorens | Institute for Molecular Medicine Finland (FIMM), HiLIFE, University of Helsinki, Helsinki, Finland | vincent.llorens@helsinki.fi | **FinnGen Analysis working group** | **FinnGen Analysis working group** |
| Hannele Laivuori | Institute for Molecular Medicine Finland (FIMM), HiLIFE, University of Helsinki, Helsinki, Finland | hannele.laivuori@helsinki.fi | **FinnGen Analysis working group** | **FinnGen Analysis working group** |
| Taru Tukiainen | Institute for Molecular Medicine Finland (FIMM), HiLIFE, University of Helsinki, Helsinki, Finland | taru.tukiainen@helsinki.fi | **FinnGen Analysis working group** | **FinnGen Analysis working group** |
| Mary Pat Reeve | Institute for Molecular Medicine Finland (FIMM), HiLIFE, University of Helsinki, Helsinki, Finland | mary.reeve@helsinki.fi | **FinnGen Analysis working group** | **FinnGen Analysis working group** |
| Henrike Heyne | Institute for Molecular Medicine Finland (FIMM), HiLIFE, University of Helsinki, Helsinki, Finland | hheyne@broadinstitute.org | **FinnGen Analysis working group** | **FinnGen Analysis working group** |
| Nina Mars | Institute for Molecular Medicine Finland (FIMM), HiLIFE, University of Helsinki, Helsinki, Finland | nina.mars@helsinki.fi | **FinnGen Analysis working group** | **FinnGen Analysis working group** |
| Joel Rämö | Institute for Molecular Medicine Finland (FIMM), HiLIFE, University of Helsinki, Helsinki, Finland | joel.ramo@helsinki.fi | **FinnGen Analysis working group** | **FinnGen Analysis working group** |
| Elmo Saarentaus | Institute for Molecular Medicine Finland (FIMM), HiLIFE, University of Helsinki, Helsinki, Finland | elmo.saarentaus@helsinki.fi | **FinnGen Analysis working group** | **FinnGen Analysis working group** |
| Hanna Ollila | Institute for Molecular Medicine Finland (FIMM), HiLIFE, University of Helsinki, Helsinki, Finland | hanna.m.ollila@helsinki.fi | **FinnGen Analysis working group** | **FinnGen Analysis working group** |
| Rodos Rodosthenous | Institute for Molecular Medicine Finland (FIMM), HiLIFE, University of Helsinki, Helsinki, Finland | rodos.rodosthenous@helsinki.fi | **FinnGen Analysis working group** | **FinnGen Analysis working group** |
| Satu Strausz | Institute for Molecular Medicine Finland (FIMM), HiLIFE, University of Helsinki, Helsinki, Finland | satu.strausz@helsinki.fi | **FinnGen Analysis working group** | **FinnGen Analysis working group** |
| Tuula Palotie | University of Helsinki and Hospital District of Helsinki and Uusimaa, Helsinki, Finland | tuula.palotie@helsinki.fi | **FinnGen Analysis working group** | **FinnGen Analysis working group** |
| Kimmo Palin | University of Helsinki, Helsinki, Finland | kimmo.palin@helsinki.fi | **FinnGen Analysis working group** | **FinnGen Analysis working group** |
| Javier Garcia-Tabuenca | University of Tampere, Tampere, Finland | javier.graciatabuenca@tuni.fi | **FinnGen Analysis working group** | **FinnGen Analysis working group** |
| Harri Siirtola | University of Tampere, Tampere, Finland | harri.siirtola@tuni.fi | **FinnGen Analysis working group** | **FinnGen Analysis working group** |
| Tuomo Kiiskinen | Institute for Molecular Medicine Finland (FIMM), HiLIFE, University of Helsinki, Helsinki, Finland | tuomo.kiiskinen@helsinki.fi | **FinnGen Analysis working group** | **FinnGen Analysis working group** |
| Jiwoo Lee | Institute for Molecular Medicine Finland (FIMM), HiLIFE, University of Helsinki, Helsinki, Finland; Broad Institute, Cambridge, MA, United States | jiwoo.lee@helsinki.fi | **FinnGen Analysis working group** | **FinnGen Analysis working group** |
| Kristin Tsuo | Institute for Molecular Medicine Finland (FIMM), HiLIFE, University of Helsinki, Helsinki, Finland; Broad Institute, Cambridge, MA, United States | kristintsuo@fas.harvard.edu | **FinnGen Analysis working group** | **FinnGen Analysis working group** |
| Amanda Elliott | Institute for Molecular Medicine Finland (FIMM), HiLIFE, University of Helsinki, Helsinki, Finland; Broad Institute, Cambridge, MA, USA and Massachusetts General Hospital, Boston, MA, USA | aelliott@broadinstitute.org | **FinnGen Analysis working group** | **FinnGen Analysis working group** |
| Kati Kristiansson | THL Biobank / Finnish Institute for Health and Welfare (THL), Helsinki, Finland | kati.kristiansson@thl.fi | **FinnGen Analysis working group** | **FinnGen Analysis working group** |
| Mikko Arvas | Finnish Red Cross Blood Service / Finnish Hematology Registry and Clinical Biobank, Helsinki, Finland | mikko.arvas@veripalvelu.fi | **FinnGen Analysis working group** | **FinnGen Analysis working group** |
| Kati Hyvärinen | Finnish Red Cross Blood Service, Helsinki, Finland | kati.hyvarinen@veripalvelu.fi | **FinnGen Analysis working group** | **FinnGen Analysis working group** |
| Jarmo Ritari | Finnish Red Cross Blood Service, Helsinki, Finland | jarmo.ritari@veripalvelu.fi | **FinnGen Analysis working group** | **FinnGen Analysis working group** |
| Olli Carpén | Helsinki Biobank / Helsinki University and Hospital District of Helsinki and Uusimaa, Helsinki | olli.carpen@helsinki.fi | **FinnGen Analysis working group** | **FinnGen Analysis working group** |
| Johannes Kettunen | Northern Finland Biobank Borealis / University of Oulu / Northern Ostrobothnia Hospital District, Oulu, Finland | johannes.kettunen@oulu.fi | **FinnGen Analysis working group** | **FinnGen Analysis working group** |
| Katri Pylkäs | University of Oulu, Oulu, Finland | katri.pylkas@oulu.fi | **FinnGen Analysis working group** | **FinnGen Analysis working group** |
| Eeva Sliz | University of Oulu, Oulu, Finland | eeva.sliz@oulu.fi | **FinnGen Analysis working group** | **FinnGen Analysis working group** |
| Minna Karjalainen | University of Oulu, Oulu, Finland | minna.k.karjalainen@oulu.fi | **FinnGen Analysis working group** | **FinnGen Analysis working group** |
| Tuomo Mantere | Northern Finland Biobank Borealis / University of Oulu / Northern Ostrobothnia Hospital District, Oulu, Finland | tuomo.mantere@oulu.fi | **FinnGen Analysis working group** | **FinnGen Analysis working group** |
| Eeva Kangasniemi | Finnish Clinical Biobank Tampere / University of Tampere / Pirkanmaa Hospital District, Tampere, Finland | eeva.kangasniemi@pshp.fi | **FinnGen Analysis working group** | **FinnGen Analysis working group** |
| Sami Heikkinen | University of Eastern Finland, Kuopio, Finland | sami.heikkinen@uef.fi | **FinnGen Analysis working group** | **FinnGen Analysis working group** |
| Arto Mannermaa | Biobank of Eastern Finland / University of Eastern Finland / Northern Savo Hospital District, Kuopio, Finland | arto.mannermaa@uef.fi | **FinnGen Analysis working group** | **FinnGen Analysis working group** |
| Eija Laakkonen | University of Jyväskylä, Jyväskylä, Finland | eija.k.laakkonen@jyu.fi | **FinnGen Analysis working group** | **FinnGen Analysis working group** |
| Nina Pitkänen | Auria Biobank / University of Turku / Hospital District of Southwest Finland, Turku, Finland | Niina.Pitkanen@tyks.fi | **FinnGen Analysis working group** | **FinnGen Analysis working group** |
| Samuel Lessard | Translational Sciences, Sanofi R&D, Framingham, MA, USA | samuel.lessard@sanofi.com | **FinnGen Analysis working group** | **FinnGen Analysis working group** |
| Clément Chatelain | Translational Sciences, Sanofi R&D, Framingham, MA, USA | clement.chatelain@sanofi.com | **FinnGen Analysis working group** | **FinnGen Analysis working group** |
| Lila Kallio | Auria Biobank / University of Turku / Hospital District of Southwest Finland, Turku, Finland | Lila.Kallio@tyks.fi | **Biobank directors** | **Biobank directors** |
| Tiina Wahlfors | THL Biobank / Finnish Institute for Health and Welfare (THL), Helsinki, Finland | tiina.wahlfors@thl.fi | **Biobank directors** | **Biobank directors** |
| Jukka Partanen | Finnish Red Cross Blood Service / Finnish Hematology Registry and Clinical Biobank, Helsinki, Finland | jukka.partanen@veripalvelu.fi | **Biobank directors** | **Biobank directors** |
| Eero Punkka | Helsinki Biobank / Helsinki University and Hospital District of Helsinki and Uusimaa, Helsinki | eero.punkka@hus.fi | **Biobank directors** | **Biobank directors** |
| Raisa Serpi | Northern Finland Biobank Borealis / University of Oulu / Northern Ostrobothnia Hospital District, Oulu, Finland | raisa.serpi@ppshp.fi | **Biobank directors** | **Biobank directors** |
| Sanna Siltanen | Finnish Clinical Biobank Tampere / University of Tampere / Pirkanmaa Hospital District, Tampere, Finland | sanna.siltanen@pshp.fi | **Biobank directors** | **Biobank directors** |
| Veli-Matti Kosma | Biobank of Eastern Finland / University of Eastern Finland / Northern Savo Hospital District, Kuopio, Finland | veli-matti.kosma@uef.fi | **Biobank directors** | **Biobank directors** |
| Teijo Kuopio | Central Finland Biobank / University of Jyväskylä / Central Finland Health Care District, Jyväskylä, Finland | teijo.kuopio@ksshp.fi | **Biobank directors** | **Biobank directors** |
| Anu Jalanko | Institute for Molecular Medicine Finland (FIMM), HiLIFE, University of Helsinki, Helsinki, Finland | anu.jalanko@helsinki.fi | **FinnGen Teams** | **Administration** |
| Huei-Yi Shen | Institute for Molecular Medicine Finland (FIMM), HiLIFE, University of Helsinki, Helsinki, Finland | huei-yi.shen@helsinki.fi | **FinnGen Teams** | **Administration** |
| Risto Kajanne | Institute for Molecular Medicine Finland (FIMM), HiLIFE, University of Helsinki, Helsinki, Finland | risto.kajanne@helsinki.fi | **FinnGen Teams** | **Administration** |
| Mervi Aavikko | Institute for Molecular Medicine Finland (FIMM), HiLIFE, University of Helsinki, Helsinki, Finland | mervi.aavikko@helsinki.fi | **FinnGen Teams** | **Administration** |
| Helen Cooper | Institute for Molecular Medicine Finland (FIMM), HiLIFE, University of Helsinki, Helsinki, Finland | helen.cooper@helsinki.fi | **FinnGen Teams** | **Administration** |
| Denise Öller | Institute for Molecular Medicine Finland (FIMM), HiLIFE, University of Helsinki, Helsinki, Finland | denise.oller@helsinki.fi | **FinnGen Teams** | **Administration** |
| Rasko Leinonen | Institute for Molecular Medicine Finland (FIMM), HiLIFE, University of Helsinki, Helsinki, Finland; European Molecular Biology Laboratory, European Bioinformatics Institute, Cambridge, UK | rasko@ebi.ac.uk | **FinnGen Teams** | **Administration** |
| Henna Palin | Finnish Clinical Biobank Tampere / University of Tampere / Pirkanmaa Hospital District, Tampere, Finland | henna.palin@pshp.fi | **FinnGen Teams** | **Administration** |
| Malla-Maria Linna | Helsinki Biobank / Helsinki University and Hospital District of Helsinki and Uusimaa, Helsinki | malla-maria.linna@hus.fi | **FinnGen Teams** | **Administration** |
| Mitja Kurki | Institute for Molecular Medicine Finland (FIMM), HiLIFE, University of Helsinki, Helsinki, Finland; Broad Institute, Cambridge, MA, United States | mkurki@broadinstitute.org | **FinnGen Teams** | **Analysis** |
| Juha Karjalainen | Institute for Molecular Medicine Finland (FIMM), HiLIFE, University of Helsinki, Helsinki, Finland | juha.karjalainen@helsinki.fi | **FinnGen Teams** | **Analysis** |
| Pietro Della Briotta Parolo | Institute for Molecular Medicine Finland (FIMM), HiLIFE, University of Helsinki, Helsinki, Finland | pietro.dellabriottaparolo@helsinki.fi | **FinnGen Teams** | **Analysis** |
| Arto Lehisto | Institute for Molecular Medicine Finland (FIMM), HiLIFE, University of Helsinki, Helsinki, Finland | arto.lehisto@helsinki.fi | **FinnGen Teams** | **Analysis** |
| Juha Mehtonen | Institute for Molecular Medicine Finland (FIMM), HiLIFE, University of Helsinki, Helsinki, Finland | juha.mehtonen@helsinki.fi | **FinnGen Teams** | **Analysis** |
| Wei Zhou | Broad Institute, Cambridge, MA, United States | wzhou@broadinstitute.org | **FinnGen Teams** | **Analysis** |
| Masahiro Kanai | Broad Institute, Cambridge, MA, United States | mkanai@broadinstitute.org | **FinnGen Teams** | **Analysis** |
| Mutaamba Maasha | Broad Institute, Cambridge, MA, United States | mmaasha@broadinstitute.org | **FinnGen Teams** | **Analysis** |
| Zhili Zheng | Broad Institute, Cambridge, MA, United States | zhengzhi@broadinstitute.org | **FinnGen Teams** | **Analysis** |
| Hannele Laivuori | Institute for Molecular Medicine Finland (FIMM), HiLIFE, University of Helsinki, Helsinki, Finland | hannele.laivuori@helsinki.fi | **FinnGen Teams** | **Clinical Endpoint Development** |
| Aki Havulinna | Institute for Molecular Medicine Finland (FIMM), HiLIFE, University of Helsinki, Helsinki, Finland; Finnish Institute for Health and Welfare (THL), Helsinki, Finland | aki.havulinna@helsinki.fi | **FinnGen Teams** | **Clinical Endpoint Development** |
| Susanna Lemmelä | Institute for Molecular Medicine Finland (FIMM), HiLIFE, University of Helsinki, Helsinki, Finland | susanna.lemmela@helsinki.fi | **FinnGen Teams** | **Clinical Endpoint Development** |
| Tuomo Kiiskinen | Institute for Molecular Medicine Finland (FIMM), HiLIFE, University of Helsinki, Helsinki, Finland | tuomo.kiiskinen@helsinki.fi | **FinnGen Teams** | **Clinical Endpoint Development** |
| L. Elisa Lahtela | Institute for Molecular Medicine Finland (FIMM), HiLIFE, University of Helsinki, Helsinki, Finland | laura.lahtela@helsinki.fi | **FinnGen Teams** | **Clinical Endpoint Development** |
| Mari Kaunisto | Institute for Molecular Medicine Finland (FIMM), HiLIFE, University of Helsinki, Helsinki, Finland | mari.kaunisto@helsinki.fi | **FinnGen Teams** | **Communication** |
| Elina Kilpeläinen | Institute for Molecular Medicine Finland (FIMM), HiLIFE, University of Helsinki, Helsinki, Finland | elina.kilpelainen@helsinki.fi | **FinnGen Teams** | **E-Science** |
| Timo P. Sipilä | Institute for Molecular Medicine Finland (FIMM), HiLIFE, University of Helsinki, Helsinki, Finland | timo.p.sipila@helsinki.fi | **FinnGen Teams** | **E-Science** |
| Oluwaseun Alexander Dada | Institute for Molecular Medicine Finland (FIMM), HiLIFE, University of Helsinki, Helsinki, Finland | alexander.dada@helsinki.fi | **FinnGen Teams** | **E-Science** |
| Awaisa Ghazal | Institute for Molecular Medicine Finland (FIMM), HiLIFE, University of Helsinki, Helsinki, Finland | awaisa.ghazal@helsinki.fi | **FinnGen Teams** | **E-Science** |
| Anastasia Kytölä | Institute for Molecular Medicine Finland (FIMM), HiLIFE, University of Helsinki, Helsinki, Finland | anastasia.shcherban@helsinki.fi | **FinnGen Teams** | **E-Science** |
| Rigbe Weldatsadik | Institute for Molecular Medicine Finland (FIMM), HiLIFE, University of Helsinki, Helsinki, Finland | rigbe.weldatsadik@helsinki.fi | **FinnGen Teams** | **E-Science** |
| Sanni Ruotsalainen | Institute for Molecular Medicine Finland (FIMM), HiLIFE, University of Helsinki, Helsinki, Finland | sanni.ruotsalainen@helsinki.fi | **FinnGen Teams** | **E-Science** |
| Kati Donner | Institute for Molecular Medicine Finland (FIMM), HiLIFE, University of Helsinki, Helsinki, Finland | kati.donner@helsinki.fi | **FinnGen Teams** | **Genotyping** |
| Timo P. Sipilä | Institute for Molecular Medicine Finland (FIMM), HiLIFE, University of Helsinki, Helsinki, Finland | timo.p.sipila@helsinki.fi | **FinnGen Teams** | **Genotyping** |
| Anu Loukola | Helsinki Biobank / Helsinki University and Hospital District of Helsinki and Uusimaa, Helsinki | anu.loukola@hus.fi | **FinnGen Teams** | **Sample Collection Coordination** |
| Päivi Laiho | THL Biobank / Finnish Institute for Health and Welfare (THL), Helsinki, Finland | paivi.laiho@thl.fi | **FinnGen Teams** | **Sample Logistics** |
| Tuuli Sistonen | THL Biobank / Finnish Institute for Health and Welfare (THL), Helsinki, Finland | tuuli.sistonen@thl.fi | **FinnGen Teams** | **Sample Logistics** |
| Essi Kaiharju | THL Biobank / Finnish Institute for Health and Welfare (THL), Helsinki, Finland | essi.kaiharju@thl.fi | **FinnGen Teams** | **Sample Logistics** |
| Markku Laukkanen | THL Biobank / Finnish Institute for Health and Welfare (THL), Helsinki, Finland | markku.laukkanen@thl.fi | **FinnGen Teams** | **Sample Logistics** |
| Elina Järvensivu | THL Biobank / Finnish Institute for Health and Welfare (THL), Helsinki, Finland | elina.jarvensivu@thl.fi | **FinnGen Teams** | **Sample Logistics** |
| Sini Lähteenmäki | THL Biobank / Finnish Institute for Health and Welfare (THL), Helsinki, Finland | sini.lahteenmaki@thl.fi | **FinnGen Teams** | **Sample Logistics** |
| Lotta Männikkö | THL Biobank / Finnish Institute for Health and Welfare (THL), Helsinki, Finland | lotta.mannikko@thl.fi | **FinnGen Teams** | **Sample Logistics** |
| Regis Wong | THL Biobank / Finnish Institute for Health and Welfare (THL), Helsinki, Finland | regis.wong@thl.fi | **FinnGen Teams** | **Sample Logistics** |
| Auli Toivola | THL Biobank / Finnish Institute for Health and Welfare (THL), Helsinki, Finland | auli.toivola@thl.fi | **FinnGen Teams** | **Sample Logistics** |
| Minna Brunfeldt | THL Biobank / Finnish Institute for Health and Welfare (THL), Helsinki, Finland | minna.brunfeldt@thl.fi | **FinnGen Teams** | **Registry Data Operations** |
| Hannele Mattsson | THL Biobank / Finnish Institute for Health and Welfare (THL), Helsinki, Finland | hannele.mattsson@thl.fi | **FinnGen Teams** | **Registry Data Operations** |
| Kati Kristiansson | THL Biobank / Finnish Institute for Health and Welfare (THL), Helsinki, Finland | kati.kristiansson@thl.fi | **FinnGen Teams** | **Registry Data Operations** |
| Susanna Lemmelä | Institute for Molecular Medicine Finland (FIMM), HiLIFE, University of Helsinki, Helsinki, Finland | susanna.lemmela@helsinki.fi | **FinnGen Teams** | **Registry Data Operations** |
| Sami Koskelainen | THL Biobank / Finnish Institute for Health and Welfare (THL), Helsinki, Finland | sami.koskelainen@thl.fi | **FinnGen Teams** | **Registry Data Operations** |
| Tero Hiekkalinna | THL Biobank / Finnish Institute for Health and Welfare (THL), Helsinki, Finland | tero.hiekkalinna@helsinki.fi | **FinnGen Teams** | **Registry Data Operations** |
| Teemu Paajanen | THL Biobank / Finnish Institute for Health and Welfare (THL), Helsinki, Finland | teemu.paajanen@thl.fi | **FinnGen Teams** | **Registry Data Operations** |
| Priit Palta | Institute for Molecular Medicine Finland (FIMM), HiLIFE, University of Helsinki, Helsinki, Finland | priit.palta@helsinki.fi | **FinnGen Teams** | **Sequencing Informatics** |
| Shuang Luo | Institute for Molecular Medicine Finland (FIMM), HiLIFE, University of Helsinki, Helsinki, Finland | shuang.luo@helsinki.fi | **FinnGen Teams** | **Sequencing Informatics** |
| Tarja Laitinen | Pirkanmaa Hospital District, Tampere, Finland | tarja.laitinen@pshp.fi | **FinnGen Teams** | **Trajectory** |
| Mary Pat Reeve | Institute for Molecular Medicine Finland (FIMM), HiLIFE, University of Helsinki, Helsinki, Finland | mary.reeve@helsinki.fi | **FinnGen Teams** | **Trajectory** |
| Shanmukha Sampath Padmanabhuni | Institute for Molecular Medicine Finland (FIMM), HiLIFE, University of Helsinki, Helsinki, Finland | sam.padmanabhuni@helsinki.fi | **FinnGen Teams** | **Trajectory** |
| Marianna Niemi | University of Tampere, Tampere, Finland | marianna.niemi@tuni.fi | **FinnGen Teams** | **Trajectory** |
| Harri Siirtola | University of Tampere, Tampere, Finland | harri.siirtola@tuni.fi | **FinnGen Teams** | **Trajectory** |
| Javier Gracia-Tabuenca | University of Tampere, Tampere, Finland | javier.graciatabuenca@tuni.fi | **FinnGen Teams** | **Trajectory** |
| Mika Helminen | University of Tampere, Tampere, Finland | mika.helminen@tuni.fi | **FinnGen Teams** | **Trajectory** |
| Tiina Luukkaala | University of Tampere, Tampere, Finland | tiina.luukkaala@tuni.fi | **FinnGen Teams** | **Trajectory** |
| Iida Vähätalo | University of Tampere, Tampere, Finland | iida.vahatalo@epshp.fi | **FinnGen Teams** | **Trajectory** |
| Jyrki Tammerluoto | Institute for Molecular Medicine Finland (FIMM), HiLIFE, University of Helsinki, Helsinki, Finland | jyrki.tammerluoto@helsinki.fi | **FinnGen Teams** | **Data protection officer** |
| Marco Hautalahti | Finnish Biobank Cooperative - FINBB | marco.hautalahti@finbb.fi | **FinnGen Teams** | **FINBB - Finnish biobank cooperative** |
| Johanna Mäkelä | Finnish Biobank Cooperative - FINBB | johanna.makela@finbb.fi | **FinnGen Teams** | **FINBB - Finnish biobank cooperative** |
| Sarah Smith | Finnish Biobank Cooperative - FINBB | sarah.smith@finbb.fi | **FinnGen Teams** | **FINBB - Finnish biobank cooperative** |
| Tom Southerington | Finnish Biobank Cooperative - FINBB | tom.southerington@finbb.fi | **FinnGen Teams** | **FINBB - Finnish biobank cooperative** |
| Petri Lehto | Finnish Biobank Cooperative - FINBB | petri.lehto@finbb.fi | **FinnGen Teams** | **FINBB - Finnish biobank cooperative** |
